# The importance of B cells for osteogenic homeostasis

**DOI:** 10.1186/s12964-026-03128-z

**Published:** 2026-08-08

**Authors:** Radost A. Saß, Christian H. Bucher, Sabine Bartosch, Il-Kang Na, Olaf Penack, Georg N. Duda, Katharina Schmidt-Bleek

**Affiliations:** 1 Julius Wolff Institut - Center for Musculoskeletal Biomechanics and Regeneration, Berlin Institut of Health at Charité— Universitätsmedizin Berlin, Berlin, Germany; 2 Berlin Institute of Health Center for Regenerative Therapies, Berlin Institute of Health at Charité— Universitätsmedizin Berlin, Berlin, Germany; 3 Nuffield Department of Orthopeadics, Rheumatology and Musculosceletal Sciences, University of Oxford, Oxford, UK; 4 Department of Hematology, Oncology and Tumor Immunology, Charité— Universitätsmedizin Berlin, Berlin, Germany

**Keywords:** Mesenchymal stromal cell, Cell differentiation, Immune modulation, Immune cells, B cells, Immune modulatory therapies, Mixed lymphocyte reaction

## Abstract

Therapeutic interventions for diseases such as leukemia and autoimmune disorders are increasingly designed to selectively target and deplete specific immune cell subsets over prolonged periods. This can disrupt the homeostasis of the adaptive immune system, with consequences not only for peripheral blood but also for the “cradle of the immune system”, the bone marrow. This study hypothesized that such immune imbalance due to immune cell depletion therapies impairs the differentiation capacity of mesenchymal stromal cells (MSCs), which are key progenitors of bone cells and are thus essential for maintaining bone health. To validate this hypothesis, a series of cell culture experiments were conducted in which MSCs were stimulated with immune-conditioned media derived from various immune cell subsets. A comprehensive analytical approach was employed to evaluate the differentiating MSCs, including their associated supernatants, deposited collagen, mineralized matrix, and lipid deposition at defined time points during their maturation. Therefore, fluorescence and nonfluorescent histological staining, enzyme-linked immunosorbent assays (ELISAs), and expression analyses with Ribonucleic acid (RNA) were performed. This study demonstrated that, compared with immune-conditioned media from T cells or peripheral blood mononuclear cells (PBMCs), B cell-immune-conditioned media enriched with interleukin 4, bone morphogenetic protein 2 and Dickkopf 1 enhanced the osteogenic differentiation of MSCs in vitro. This positive osteogenic effect is driven primarily by elevated cytokine secretion by stimulated B cells, which in vivo in turn potentially stimulate bone turnover and modulate immune system function. In summary, these data demonstrate an pro-osteogenic and pro-adipogenic influence of B cells on differentiating MSCs in vitro, suggesting that an imbalance in immune cells in the bone marrow perturbs bone homeostasis.

**Supplementary Information:**

The online version contains supplementary material available at 10.1186/s12964-026-03128-z.

## Introduction

Bones are essential components of the musculoskeletal system and a primary lymphatic organ, highlighting the close interconnection between the immune and skeletal systems. Bones are home to hematopoietic stem cells (HSCs) and immune precursor cells. Osteoimmunology, a relatively new interdisciplinary field of research, has revealed the dynamic interactions between the skeletal and immune systems. These interactions are crucial for maintaining skeletal tissue homeostasis and for the pathogenesis of immune and skeletal diseases [1, 2]. Abnormal immune activation can stimulate bone cells, such as osteoclasts and osteoblasts, disrupt bone remodeling and potentially lead to skeletal diseases. Conversely, a complex multicellular network in the bone marrow creates a specialized microenvironment that is essential for maintaining and differentiating HSCs and their progeny. Dysregulation of this intricate interplay occurs during myeloablative therapies in preparation for bone marrow transplantation, for example. Minor alterations in cell density or the absence of particular cell types can significantly influence an organism’s overall regenerative capacity. The initial examination focused on the role of the innate immune system in the regenerative process of bone, wherein it stimulated the differentiation of osteoprogenitor cells into osteoclasts. This process was found to be essential for the clearance of damaged tissue and the preparation of the site for the subsequent activity of osteoblasts, which are responsible for the formation of new bone. Subsequent research demonstrated that T cells could also induce the formation of osteoclasts, thereby establishing the field of osteoimmunology. This newfound knowledge expanded the study of the adaptive immune system to include its impact on regenerative processes [3–7].

Following pathological events, bone is one of the few tissues capable of complete regeneration. This includes scar-free healing after fractures and the reconstitution of bone marrow after myeloablative therapies such as chemotherapy and radiotherapy in preparation for stem cell transplantation. Successful regeneration depends on a well-coordinated interaction between stromal and immune cells within the bone microenvironment, which is an essential prerequisite for the formation of the necessary environment for immune and bone cell differentiation within bone marrow niches. Recent studies have demonstrated the critical role of adipocytes in maintaining bone homeostasis and facilitating regeneration [8]. Their close interconnectivity with vessel and osteogenic cells, as evidenced in recent research, underscores their significance in bone biology [9]. Consequently, the adipogenic differentiation capacity of stromal cells has become a focal point in the field of regenerative medicine.

Myeloablative therapies, which include radiotherapy, chemotherapy and total body irradiation, result in destruction of the bone marrow structure as well as the bone marrow niches. Bone marrow reconstitution is essential for restoring bone stability and immune function in patients receiving such therapies [10]. Total body irradiation has been demonstrated to result in decreased bone mass and an elevated risk of osteoporosis [11–13] indicating that the reconstitution of the bone marrow environment requires time and that during this time, disturbed immune-bone interplay causes bone loss. Previously, we showed in a preclinical model recapitulating stem cell transplantation that the type of HSCs used during transplantation also influences the time-dependent reconstitution of bone and bone marrow, as well as the restoration of immune cell populations within the bone marrow [3]. Analyses of the bone marrow composition revealed an immune imbalance that disrupted bone homeostasis. The reconstitution of bone after myeloablative therapies and hematopoietic stem cell transplantation (HSCT) requires the reconstitution of intact bone marrow niches. Bone marrow niches comprise a diverse range of cells, including mesenchymal stem/stromal cells (MSCs), endothelial cells, preosteoblasts and osteoblasts. HSCs also reside in a specialized niche of the bone marrow where they remain until maturation is induced. During maturation, HSCs migrate between distinct niches and undergo differentiation until fully mature, at which point they are released into the bloodstream. Traumatic events or treatments that disrupt the normal bone marrow structure can impair hematopoiesis, which often leads to insufficient reconstitution of the local immune cell composition [14–19].

A growing body of research has emerged in recent years, examining the impact of depletion of immune cell subtypes especially B cells on various autoimmunity diseases [20–22]. These therapies result in imbalances in the immune cells of the bone and bone marrow [22].

We hypothesized that an imbalance in adaptive immune cell compartment alters the differentiation capacity of MSCs and thereby impacts bone marrow niches. To investigate this, we examined how specific immune cell populations and changes in their proportions affect MSC osteogenic and adipogenic differentiation potential. Additionally, we aimed to elucidate the mechanisms driving these alterations in bone formation. In summary, the present findings indicate that, within the developmental phase of novel therapeutic strategies, the influence on the immune bone and, consequently, the impact on bone health should be considered.

## Materials and methods

**Fig. 1:**
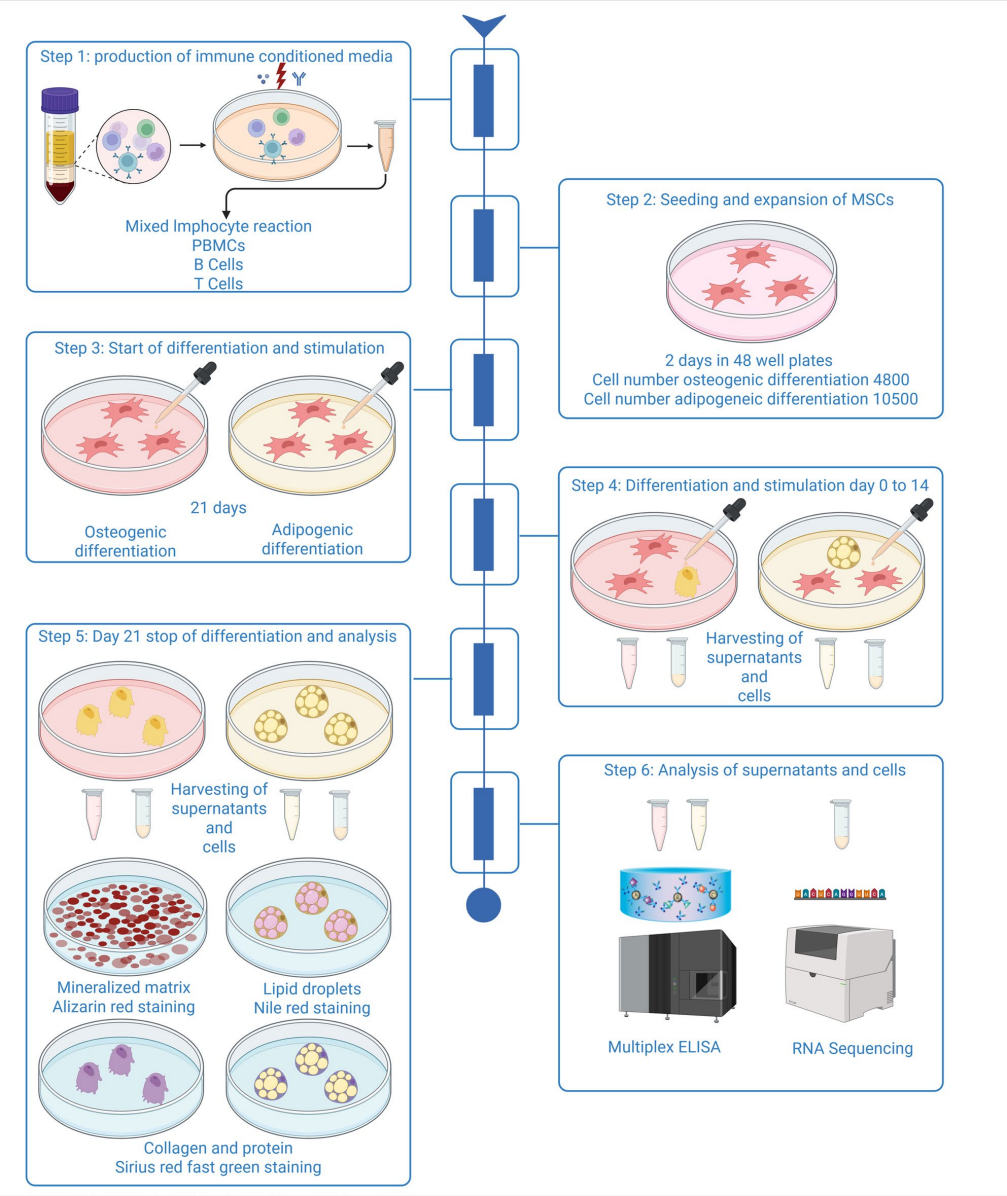
Overview of the methods used to analyze how adaptive immune cells influence MSC differentiation. Step (1) Immune-conditioned media are produced by isolating peripheral blood mononuclear cells, B cells, and T cells. These cells were then incubated with the appropriate stimuli for a designated period. Thereafter, the cell culture medium was harvested and designated immune-conditioned medium. Step (2) MSCs were seeded in 48-well plates with expansion medium. For the purpose of osteogenic differentiation, 4,800 cells were seeded and subsequently expanded for two days. For the purpose of adipogenic differentiation, 10,500 cells were similarly seeded and expanded for a period of two days. Step (3) The differentiation and stimulation process was initiated by removing the expansion medium and replacing it with osteogenic differentiation medium or adipogenic differentiation medium, along with immune-conditioned medium, which was then added to the cells. The differentiation and stimulation process was executed over a period of 21 days, with medium changes being implemented at regular intervals. Step (4) In the initial phase of differentiation (days 0 to 14), the production of both the cells and the cell culture media was monitored at predetermined intervals. These samples were then preserved for subsequent analysis. Step (5) The differentiation process was terminated after a 21-day period. The collection of cells and the subsequent extraction of the medium were conducted with the objective of preserving these materials for future analysis. The mineralized matrix, lipid droplets, and collagen were subjected to Alizarin red, Nile red, and Sirius red fast green staining. Step (6) The subsequent analysis of the stored supernatants was performed via multiplex ELISA. The cell status was analyzed at the RNA level via RNA sequencing

### Ethics

All research concerning human cells and blood samples was performed in accordance with the Declaration of Helsinki and has been approved through the local ethics committee and all patients gave written informed consent: EA1/125/10; EA099/10; EA2/089/20.

### Study design

Blood was obtained from a single healthy donor for the isolation of peripheral blood mononuclear cells (PBMCs) to create immune-conditioned media. A comprehensive characterization was conducted to assess the impact of the immune-conditioned media (ICM). The same ICM was used on MSCs from six different donors.

### Isolation and activation of immune cells

Immune-conditioned media were produced with immune cells from one donor. In order to create a cross-reactive self-stimulation of peripheral blood mononuclear cells (PBMCs), the production of mixed lymphocyte reaction (MLR) immune-conditioned medium was performed using PBMCs from a second donor. PBMCs were isolated by diluting the blood 1:2 with Phosphate-buffered saline (PBS, Gibco, Carlsbad, CA; USA) supplemented with 2% fetal bovine serum (FBS Sera low, Pan Biotech, Aidenbach, Germany) before being transferred to SepMate tubes filled with Lymphoprep (85450 and 18060, Stemcell, Vancouver, Canada). The tubes were then centrifuged at 1200 × g for 15 min at room temperature, the plasma and red blood cells were discarded, and the lymphocyte layer, containing the PBMCs, was transferred to another tube. PBMCs were washed twice with PBS and FBS when used directly for culture or once with PBS containing 10% bovine serum albumin (BSA, Sigma Aldrich, St. Louis, MO, USA) and 2 mM EDTA (Sigma Aldrich, St. Louis, MO, USA) when subjected to an additional isolation step.

B and T cells were isolated from an aliquot of PBMCs via EasySep kits (#17954 and 17951, Stemcell, Vancouver, Canada). PBMCs were first incubated with the isolation cocktail for 5 min, followed by a 5-minute incubation with magnetic particles. The sample volume was adjusted to 2.5 ml, and the target T cells were magnetically separated for 2.5 min before being transferred to a new tube, following the manufacturer’s instructions (Fig. 1 step 1). The purity of the isolates was tested with flow cytometric analysis (Figure S 1).

**Fig. 2:**
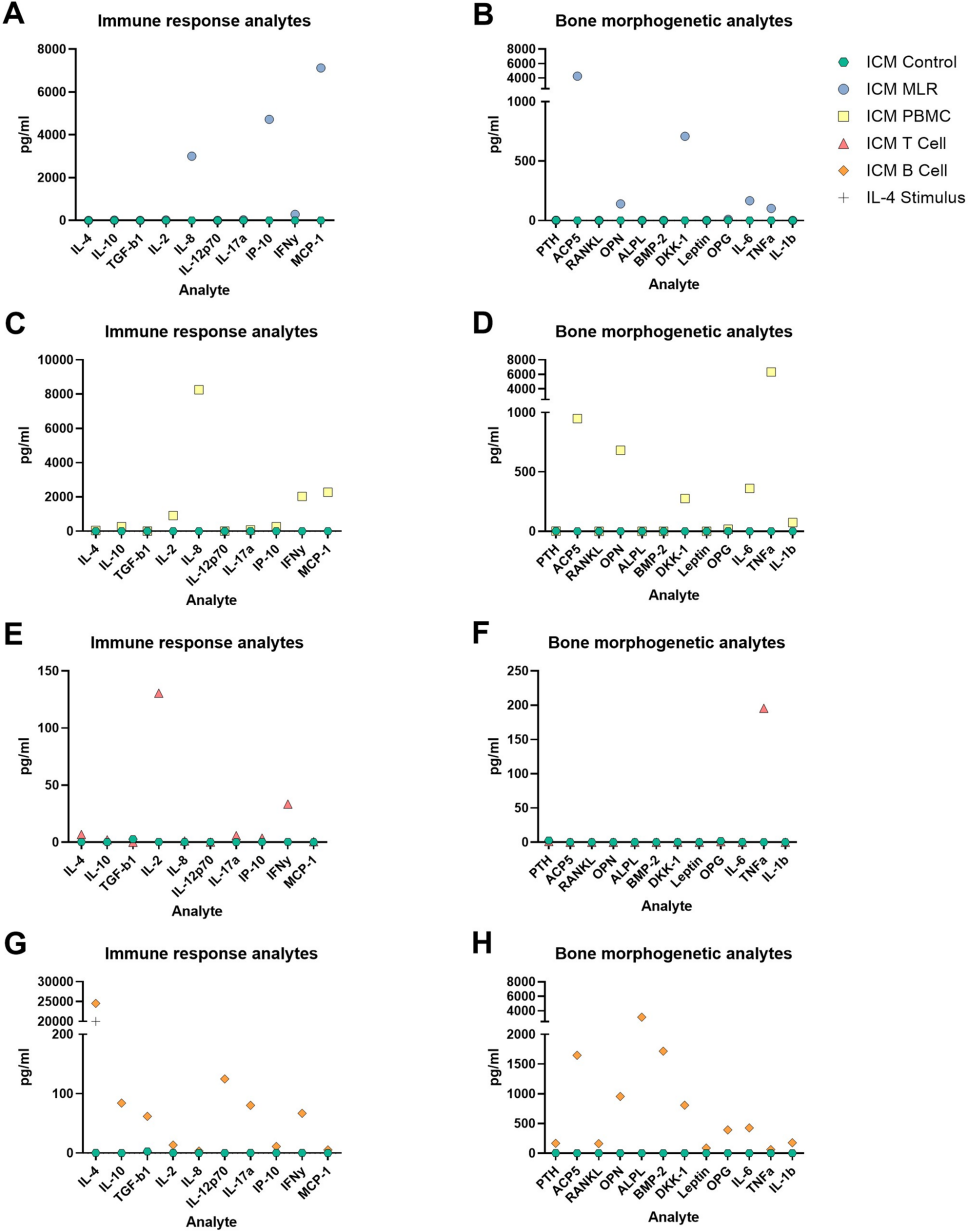
Data from multiplex ELISAs **A**-**B**) ELISA analyses of mixed lymphocyte reaction-immune-conditioned medium. **C**-**D** ELISA analyses of PBMC-immune-conditioned medium. **E**-**F** ELISA analyses of T Cell-immune-conditioned medium. **G**-**H** ELISA analyses of B cell-immune-conditioned medium ICM Control = medium control, ICM MLR = mixed lymphocyte reaction-immune-conditioned medium, ICM PBMC = activated peripheral blood mononuclear cell-immune-conditioned medium, ICM T cell = activated T cell-immune-conditioned medium, ICM B cell = activated B cell immune-conditioned medium, IL-4 Stimulus = IL-4 concentration used to activate B cells. Data were measured in technical triplets, the data points shown are the means of the triplets, *n*=1

Following their isolation from PBMCs, B and T cells are activated to generate immune-conditioned medium enriched with cytokines and proteins secreted during immune activation. This medium was used to investigate how the activated specific immune cell subset influences the differentiation capacity of MSCs. The isolated immune cells were adjusted to the desired concentration via roswell park memorial institute (RPMI) medium 1640 supplemented with 10% FBS, 1% penicillin/streptomycin (Gibco, Carlsbad, CA; USA), and 1% GlutaMax (Gibco, Carlsbad, CA; USA). Subsequently, 1 ml of the immune cell suspension was transferred to each well of a 24-well plate and stimulated with the designated agents. The cell numbers and stimulation protocols are listed in Table 1. The number of cells was determined by the percentage of T cells (50%) and B cells (10%) in PBMCs [23]. The number of PBMCs from each donor for the mixed lymphocyte reaction was selected following consultation with the Department of Immunology at the Berlin Institute of Health, an institution with expertise in mixed lymphocyte reaction experiments.

**Table 1:** Stimulation protocol and required numbers of isolated lymphocytes

Immune conditioned medium (ICM)	Cells	Cell number per ml	Stimuli	Incubation time
Mixed lymphocyte reaction (MLR) ICM	PBMCs from two donors	Donor1 660,000 Donor2 340,000	Self-stimulation	4 days
PBMC ICM	PBMCs from Donor 1	1x10^6^	Anti CD3 10 µg/ml Anti CD28 100 µg/ml	24 h
T Cell ICM	T cells from Donor 1	500,000	Anti CD3 10 µg/ml Anti CD28 100 µg/ml	24 h
B Cell ICM	B cells from Donor 1	100,000	Anti IgM 5 µg/ml IL-4 20 ng/ml	48 h

After the designated incubation time, activation was stopped by removing the 24-well plate from the incubator. The immune-conditioned media were transferred to protein LoBind tubes and centrifuged at 1000 × g for 10 min at 4 °C to remove the cell debris. The supernatant was filtered through a 0.2 μm syringe filter, aliquoted and frozen directly on dry ice. The immune-conditioned medium was stored at -80 °C until needed (Fig. 1 step 1). The quantity of activated immune cells was selected to correspond to the physiological amount of immune cell subsets with 50% T cells and 10% B cells in the subset of PBMCs and to replicate the amount of cytokines that are typically secreted from these cells during activation within the body [24]. The mixed lymphocyte reaction was established with the help from the Immunological Institute of the Charité that mirrors the reaction of graft lymphocytes to host lymphocytes after transplantation. PBMCs and T cells were stimulated using a well-established activation protocol that employed anti-CD3 and anti-CD28 antibodies. The activation of B cells was examined in experiments in preparation of this study through the use of various antibodies and cytokines in order to identify the most effective activation protocol. Each final immune-conditioned medium was produced once. The efficacy of the activation was then subjected to rigorous testing through flow cytometric analysis and the quantification of the secreted cytokines with two technical replicates (Figure S 2, Fig. 1).

### Isolation of bone marrow mesenchymal stromal cells

Bone marrow MSCs were cryopreserved and anonymized provided by the Core Unit Cell and Tissue Harvesting of the Berlin Institute of Health Center for Regenerative Therapies. Bone marrow was obtained from human donors who were undergoing hip surgery. The isolation of mesenchymal stem cells (MSCs) was performed through a density gradient separation method that utilized Histopaque-1077 (Sigma-Aldrich, St. Louis). This was followed by the adhesion of the isolated MSCs to tissue culture polystyrene. The culture medium used in this study was Dulbecco’s modified Eagle’s medium (DMEM) (Gibco, Grand Island, NY), which was supplemented with 10% fetal calf serum (FCS) (Biochrom AG, Berlin) and 100 U/mL penicillin plus 100 µg/mL streptomycin [25, 26].

### Expansion, seeding and stimulation of MSCs and osteoblasts

Before using MSCs for the experiments their stem cell properties were tested. Their capacity to undergo osteogenic and adipogenic differentiation was examined by the Core Unit. Additionally, the MSCs were evaluated for the expression of stem cell markers using flow cytometry. The MSCs used were positive for the markers CD73, CD90, CD105, CD146, CD166, CD271, STRO-1, and negative for CD14, CD 11b, CD19, CD34, CD45, HLA-DR. It is imperative to conduct a series of rigorous tests on the MSCs before their application. The necessity of such testing stems from the potential for impurities during the harvesting of the aforementioned cells. Specifically, these impurities can lead to an uncontrolled overgrowth by competing cells. Consequently, a comprehensive testing protocol must be implemented prior to the utilization of these cells. To expand the cryopreserved cells, they were rapidly thawed by transferring them from dry ice to a 37 °C water bath and then seeded into a cell culture flask at a density of 1600 cells per cm². The expansion medium consisted of low-glucose Dubelcos minimum essential medium (DMEM), 10% FBS (FBS Sera low, Pan Biotech, Aidenbach, Germany), 1% GlutaMAX (Gibco, Carlsbad, CA, USA), and 1% penicillin/streptomycin 10,000 UI/ml (Gibco, Carlsbad, CA, USA). The expansion medium was changed every two to three days until a confluence of approximately 80% was reached. Afterwards, the cells were washed with PBS and detached by adding the appropriate amount of Tryp LE Express (Gibco, Carlsbad, CA, USA) in accordance with the manufacturer’s instructions, followed by a 5 min incubation at 37 °C. For differentiation and stimulation MSCs with a doubling number of 3 or 4 were used and 4800 cells (osteogenic differentiation) or 10,500 cells (adipogenic differentiation) were seeded into each well of a 48-well plate and cultured for 48 h at 37 °C and 5% CO_2_. The cells were maintained in expansion medium consisting of DMEM enriched with 10% FBS, 1% penicillin/streptomycin, and 1% GlutaMax. After 48 h, the medium was removed from the cells and replaced with fresh expansion medium, osteogenic differentiation medium or adipogenic differentiation medium to initiate differentiation. The composition of the media is shown in Table 2. The immune-conditioned media were added to the differentiation media at a 1:4 dilution. For the control medium RPMI 1640, which was supplemented with 10% FBS, 1% penicillin/streptomycin, and 1% GlutaMax, was utilized due to its role as the medium in which the immune cells were incubated during the activation process. The culture medium was changed every three to four days, with immune-conditioned media replenished at each change. MSCs exposed to immune-conditioned media during their differentiation into adipocytes and osteoblasts are referred to as stimulated and differentiated MSCs.

**Table 2:** Composition of media used for expanding and differentiating MSCs

Medium	Supplement	Amount
Expansion medium	DMEM low Glucose	As needed
	FCS	10%
	Penicillin/Streptomycin	1%
	Glutamax	1%
Osteogenic medium	DMEM low Glucose	As needed
	Dexamethasone-Water Soluble	0,1 µM
	L-Ascorbic acid 2-phosphate sesquimagnesium salt hydrate	50 µM
	ß-Glycerolphosphate disodium salt hydrate	10 mM
	Penicillin/Streptomycin	1%
	Glutamax	1%
	FCS	10%
Adipogenic medium	DMEM High Glucose	As needed
	Dexamethasone-Water Soluble	1 µM
	Insulin	2 µM
	3-Isobutyl-1-methylxanthin	500 µM
	Indomethacin	100 µM
	Penicillin/Streptomycin	1%
	FCS	10%

### Medium collection and cell harvesting of differentiated MSCs

To analyze the effects of immune-conditioned media on MSCs at multiple levels, MSC populations from six different donors were cultivated and stimulated in three technical replicates in parallel in 48-well plates. Some analyses were performed directly. Differentiated MSCs and the corresponding supernatants were harvested and stored at -20 °C for subsequent analyses.

For cytokine and RNA analyses, culture plates were removed from the incubator, and the supernatant of the differentiated MSCs was immediately transferred to protein LoBind tubes. The supernatant was then centrifuged for 5 min at 1000 × g and transferred to new tubes. Freezing was performed directly on dry ice. In the next step, the differentiated MSCs were washed with warm PBS once and then harvested in PBS by detaching them with a cell scraper, transferred to a DNA-LoBind tube, and centrifuged for 5 min at 1000 × g to remove the supernatant. The cells were then frozen directly in dry ice. The differentiated MSCs and their supernatants were stored at -80 °C until analysis.

### Analyses

The analyses were conducted in three technical replicates. Normalization of data was performed for each technical replicate. Subsequent to the normalization process, the replicates were consolidated into a single entry. The consolidation of biological replicates was instrumental in facilitating the graphical representation of the data. This graphical representation, depicted in boxplots, delineates the minimum value, lower quartile (25%), the median (50%) the top quartile (75%) and the maximum. Quantification of cells via Hoechst staining.

The cell nuclei were directly stained with Hoechst dye in the designated 48-well plates. The cells were fixed with 4% paraformaldehyde solution for 10 min and then washed twice with PBS. One hundred microliters of Hoechst solution (0.0286 mM in distilled water) was added, and the cells were incubated in the dark at room temperature for 10 min. Afterwards, the cells were washed twice with PBS, and 150 µl of PBS was added to each well for fluorescence measurement at an excitation wavelength of 364 nm and an emission wavelength of 460 nm. To account for intrinsic plate fluorescence, PBS was added to additional wells without cells, and their fluorescence was measured. The resulting background signal was subtracted from the corresponding sample measurements.

### Analysis of adipogenic differentiation via Nile red staining

The effect of stimulation on the adipogenic differentiation of MSCs was analyzed by quantifying intracellular fat droplets via Nile red staining. For this purpose, the cells were washed twice with PBS and then incubated with 150 µl of a 0.34 µM Nile red solution in the dark at room temperature for 20 min. After staining, the cells were washed twice with PBS, and 150 µl of PBS was added to each well for fluorescence measurement (excitation: 530 nm; emission: 635 nm). Nile red fluorescence was normalized to the number of cells, as determined by Hoechst staining.

### Analysis of osteogenic differentiation via Alizarin Red staining

To determine mineralization, the cell layer in the wells was washed twice with Ampuwa and incubated with 150 µl of 1.872 M Alizarin Red solution for 10 min at room temperature. After staining, the cell layer was thoroughly washed with deionized water. For quantification, 124 µl of cetylpyridinium chloride was added to each well and incubated until the Alizarin Red was fully dissolved. The absorbance of the solution was measured at 562 nm. The Alizarin Red data were normalized to the number of cells determined by Hoechst staining.

### Analysis of collagen production via Sirius Red fast green staining

To quantify the amount of collagen produced during osteogenic differentiation a Sirius Red Fast Green staining kit (Chondrex, Redmond, WA, USA) was used to stain total protein and collagen. The cell layer was fixed with 4% paraformaldehyde solution for 10 min, washed twice with PBS, and then incubated with 100 µl of the staining solution. Following a 30-minute incubation, the cell layer was rinsed with deionized water until it appeared clear. To quantify the degree of staining, extraction buffer was added to the wells, which were pipetted up and down until the dye fully dissolved. The absorbance was measured at 540 nm for collagen quantification and at 605 nm for total protein content.

### Analysis of early adipogenic differentiation via FABP4 ELISA

The concentration of FABP4 in the collected supernatants was determined via ELISA (EH177RB, Invitrogen; Waltham, MA, USA) to investigate how stimulation affects the early phase of MSC adipogenic differentiation. The Invitrogen FABP4 ELISA was performed according to the manufacturer’s instructions. The samples were incubated for 2.5 h at room temperature on precoated plates. After washing, the biotin conjugate was added, and the mixture was incubated for 1 h, followed by another wash. Streptavidin-Horse reddish peroxidase was then added, and the mixture was incubated for 45 min. After a final washing step, the Tetramethylbenzidine substrate was applied for 30 min. The reaction was stopped, and the absorbance was measured at 450 nm via a plate reader.

### Analysis of secreted cytokines in the bone metabolism plex and immune response plex

To quantify cytokines involved in bone metabolism and the regulation of the immune response, two multiplex ELISAs (741362 and 740930, Biolegend, San Diego, CA, USA) were performed in accordance with the manufacturer’s instructions. The samples were mixed with premixed beads and incubated for 2 h. After a washing step, detection antibodies were added, and the samples were incubated for 1 h. Streptavidin-Phycoerithrin was then added, and the mixture was incubated for 30 min, followed by a final washing step. The beads were analyzed via a flow cytometer (Sony, Weybridge, UK).

### Analysis of genomic changes via RNA isolation and sequencing

Total RNA was isolated from frozen cells via a RNeasy Mini Kit (Qiagen, Hilden, Germany). As described in the manufacturer’s protocol, the cells were lysed via RLT buffer containing 0.5% 2-mercaptoethanol, and the lysate was transferred to a RNeasy spin column. The column was washed twice with RW1 buffer, and the desoxyribonuclease was incubated for 15 min between the washes. The column was subsequently washed twice with RPE buffer, and the RNA was released in RNase-free water and collected in desoxyribonucleic acid (DNA) LoBind tubes. The RNA concentration and purity were measured via a NanoDrop spectrophotometer (Implen, Munich, Germany). Before sequencing the RNA of technical replicates was pooled. The RNA samples were stored at -80 °C until sequencing, which was performed by the Core Unit Genomics of the BIH at the Charité. Data analysis was performed via R studio.

### Statistics

The data were subjected to statistical analysis via the nonparametric Mann‒Whitney test. The data are presented as box plots and heatmaps. Owing to the limited sample size, the assumption of a normal distribution was not met. The threshold for statistical significance was set at *p* ≤ 0.05.

## Results

To analyze the impact of specific cells of the adaptive immune system, primary human cells from six donors were subjected to immune-conditioned medium from activated B cells, T cells, PBMCs and from a mixed lymphocyte reaction (MLR) simulating a graft-versus-host situation. Immune cells for the conditioned immune medium were harvested from one donor. The production of each immune-conditioned medium was done once. The efficacy of the cell isolation process was evaluated through the implementation of flow cytometry. The results of this analysis are presented in Figure S 1. PBMCs showed a purity of 51.2% due to a higher amount of cell debris in the isolate. The purity of T cells and B cells was determined to be 99.5% and 99.9%, respectively. The activation of immune cells was customized according to the specific cell subset and subsequently evaluated through the implementation of cytokine secretion. In addition, flow cytometric analysis was implemented (Figure S 2). The assessment of T cell activation entailed the evaluation of changes in the proportion of CD8 + T cells and the amount of CD45RA- or CD62L-positive cells. In the context of B cells, the investigation focused on the decline of CD27 + cells.

### The competence of immune-conditioned media is determined by secreted cytokines

In this study, cell culture experiments were conducted to investigate how immune-conditioned media from differentially activated immune cells influence the differentiation of MSCs. First, cytokine levels in the immune-conditioned media were quantified to assess the efficacy of the differential immune cell activation. DKK-1, Interleukin 8, Interferon gamma-induced protein 10, Interferon gamma and Monocyte chemotactic protein 1 (IL-8, IP-10, IFNγ and MCP-1) were increased in MLR-immune-conditioned medium (Fig. 2A-B). Furthermore, PBMC-immune-conditioned medium increased the levels of acid phosphatate 5 (ACP-5), DKK-1, Osteopontin (OPN), IL-8, and interleukin 2 (IL-2, IFNγ, MCP-1 and tumor necrosis factor alpha (TNFα) (Fig. 2C-D). Consequently, the factors induced by MLR and PBMC activation create an environment characterized by elevated levels of bone resorption and inflammation. T cell-immune-conditioned medium increased the level of TNFα, IL-2 and IFNγ (Fig. 2E-F). In addition, B cell-immune-conditioned medium contained increased concentrations of cytokines such as parathormone, alkaline phosphatase, bone morphogenetic protein 2, Dickkopf 1, Leptin, Osteoprotegerin, IL6, Interleukin 1b and Interleukin 4 (PTH, ALPL, BMP-2, DKK-1, leptin, OPG, IL-1b and IL-4) (Fig. 2G-H), indicating that activated B cells secrete factors that enhance bone turnover, including bone resorption and bone formation, as well as pro and anti-inflammatory reactions. 

### Differences in the capacity for osteogenic and adipogenic differentiation of MSCs after stimulation with immune-conditioned media

To analyze the influence of the immune-conditioned media on the MSC differentiation capacity, three assays were performed: alizarin red staining to evaluate mineralization, collagen and protein staining to assess matrix production after osteogenic differentiation, and Nile red staining to analyze lipid droplets after adipogenic differentiation.

**Fig. 3:**
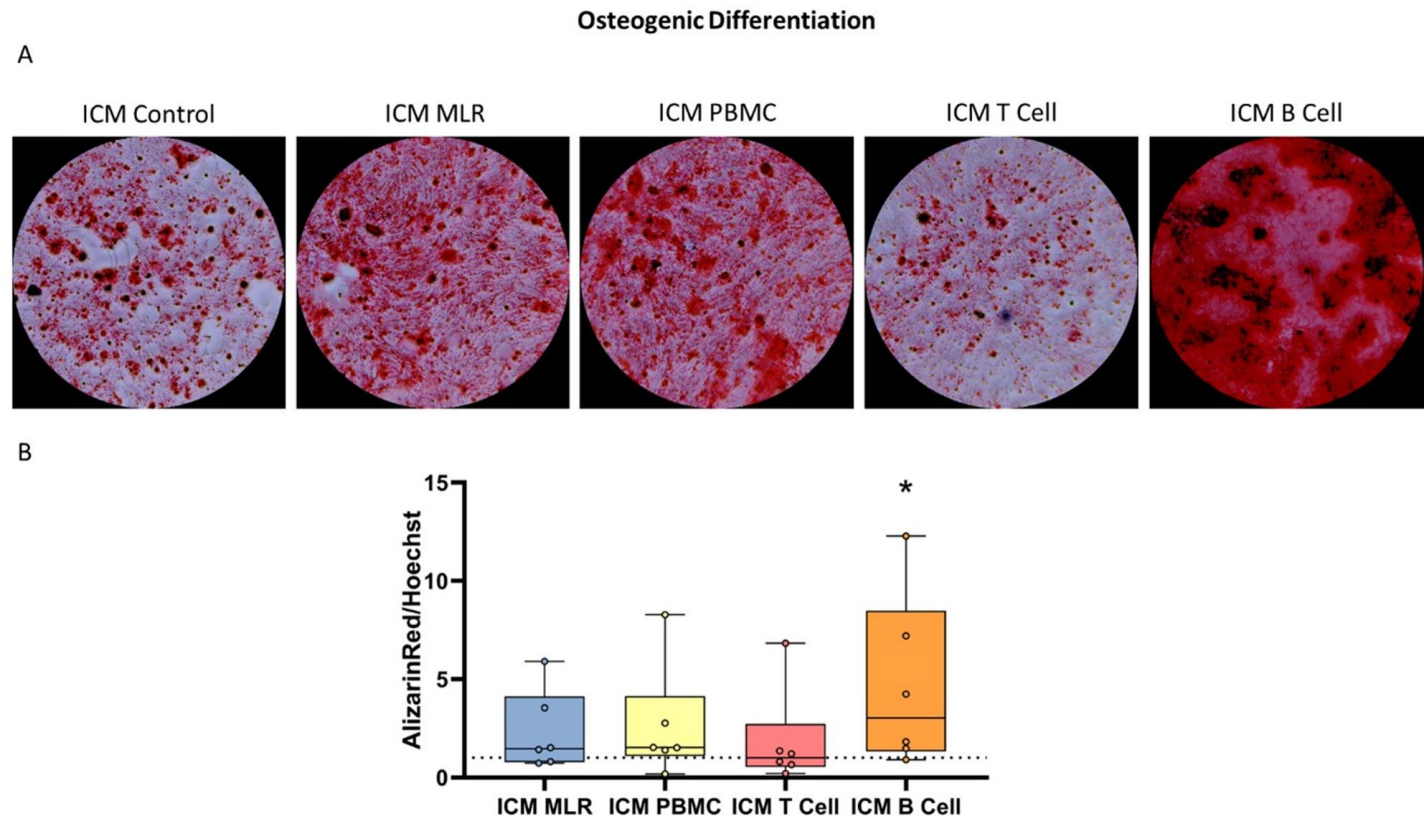
Alizarin Red staining after 21 days of osteogenic differentiation of MSCs. **A** Representative images of alizarin red staining of differentiated MSCs treated with different immune-conditioned media on day 21. **B** Quantification of alizarin red staining normalized to that of the control medium (dotted line). ICM MLR = mixed lymphocyte reaction immune-conditioned medium, ICM PBMC = activated peripheral blood mononuclear cell immune-conditioned medium, ICM T cell = activated T cell-immune-conditioned medium, ICM B cell = activated B cell immune-conditioned medium. Statistics were tested with the Mann‒Whitney test. A comparison with the control medium was conducted, and the results indicated a significant difference, as visualized with *. The boxplots show the means of the technical replicates of each donor from minimum to maximum. *n*=6, *p* value * = 0,0488

**Fig. 4:**
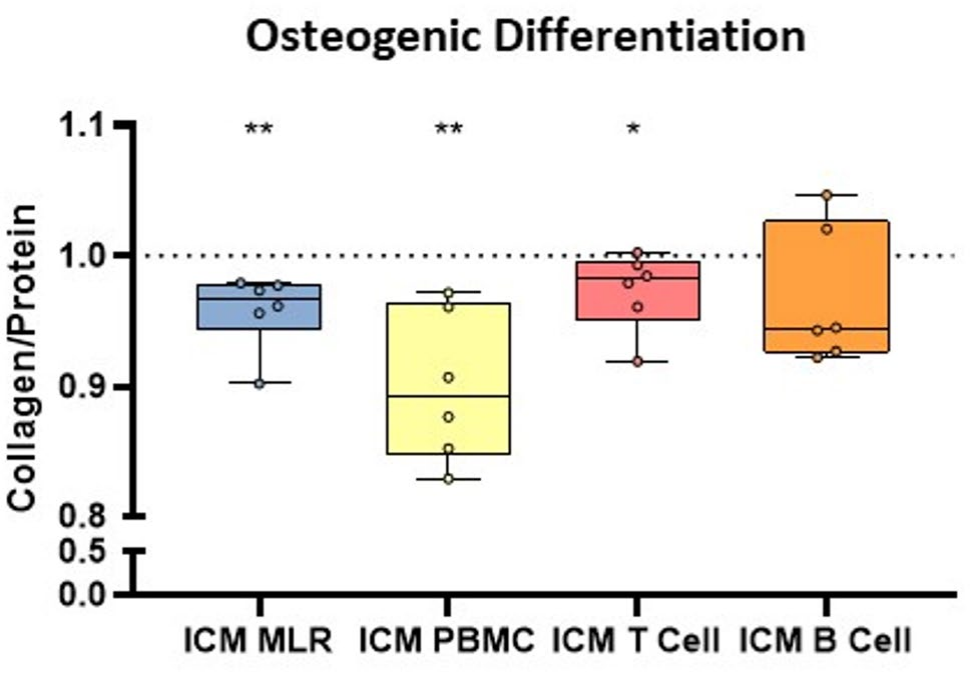
Collagen deposition after 21 days of osteogenic differentiation of MSCs. The amount of collagen was set in relation to the total cellular protein and was normalized to the amount of differentiation control. ICM MLR = mixed lymphocyte reaction immune-conditioned medium, ICM PBMC = activated peripheral blood mononuclear cell immune-conditioned medium, ICM T Cell = activated T cell immune-conditioned medium, ICM B Cell = activated B cell-immune-conditioned medium. Statistics were tested with the Mann-Whitney test. The boxplots show the means of the technical replicates of each donor from minimum to maximum *n*=6, *p* value * = 0,0476, ** = 0.0022

The stimulation of MSCs with the four different immune-conditioned media resulted in different amounts of deposited mineralized matrix during osteogenic differentiation. Stimulation with MLR-, PBMC- and T cell-immune-conditioned media resulted in no significant changes in MSC matrix deposition. In contrast, a significant increase in mineralized matrix deposition was observed in differentiating MSCs stimulated with B cell-immune-conditioned medium compared with the control (Fig. 3). The quantification of collagen production in relation to total cellular protein content revealed that MLR-, PBMC- and T cell-immune-conditioned media reduced the production of collagen during the osteogenic differentiation of MSCs, whereas B cell-immune-conditioned media did not have this negative effect (Fig. 4).

**Fig. 5:**
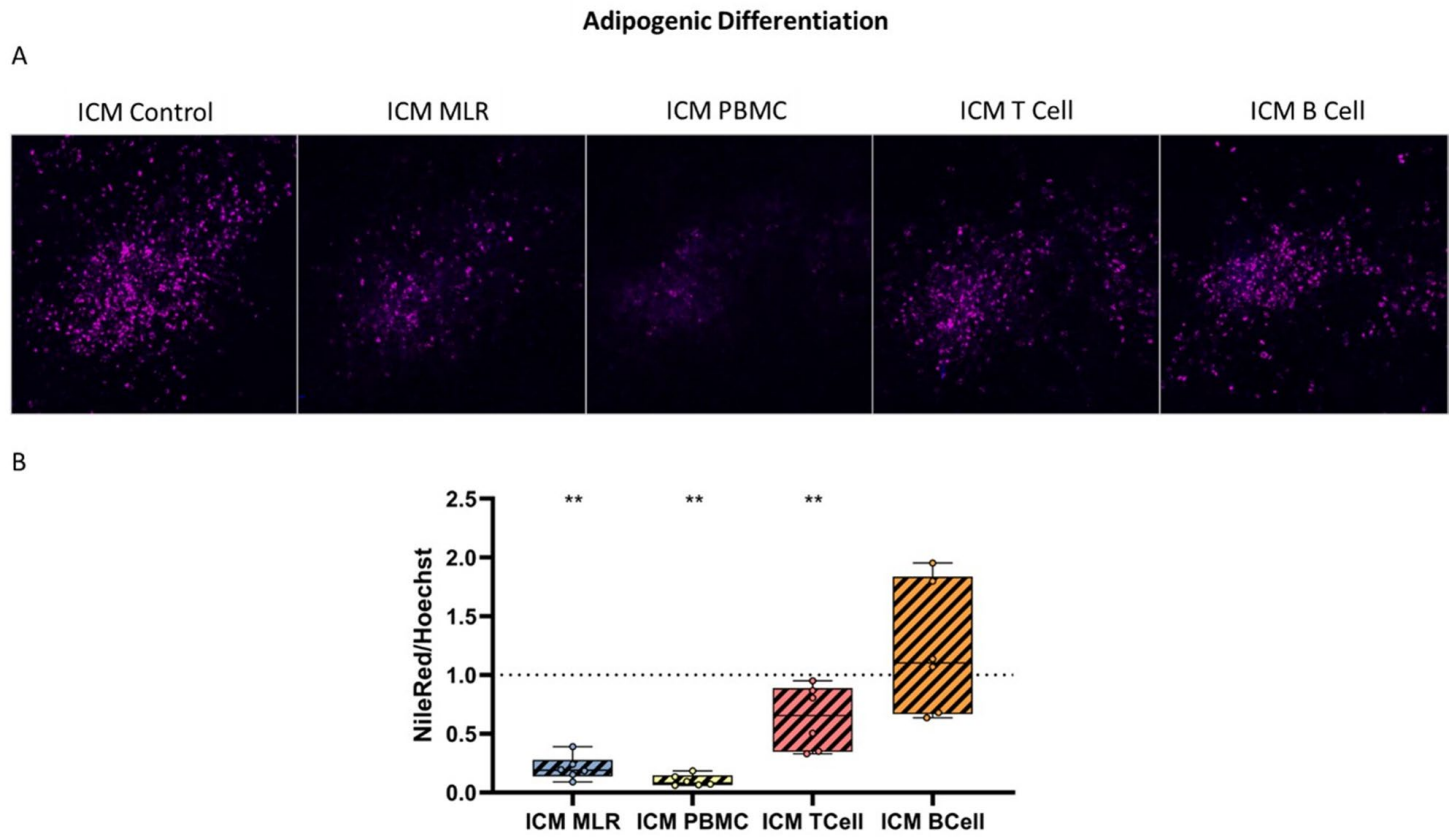
Results of adipocyte staining with Nile red. **A** Microscopy images of adipocytes derived from MSCs from one representative donor. **B** Number of adipocytes (Nile Red staining) relative to the total number of cells, as indicated by the number of Hoechst-stained nuclei. The data were normalized to the differentiation control. ICM MLR = mixed lymphocyte reaction immune-conditioned medium, ICM PBMC = activated peripheral blood mononuclear cell-conditioned medium, ICM T cell = activated T cell-conditioned medium, ICM B cell = activated B cell-conditioned medium. Statistics were tested with the Mann-Whitney test. The boxplots show the means of the technical replicates of each donor from minimum to maximum *n*=6, *p* value * = 0,0022

MSCs were induced to differentiate not only into osteogenic cells but also into adipocytes (Fig.  5). Adipocyte formation decreased after stimulation with MLR-, PBMC-, and T cell-immune-conditioned media, whereas B cell-conditioned media did not affect the adipogenic differentiation of MSCs.

**Fig. 6:**
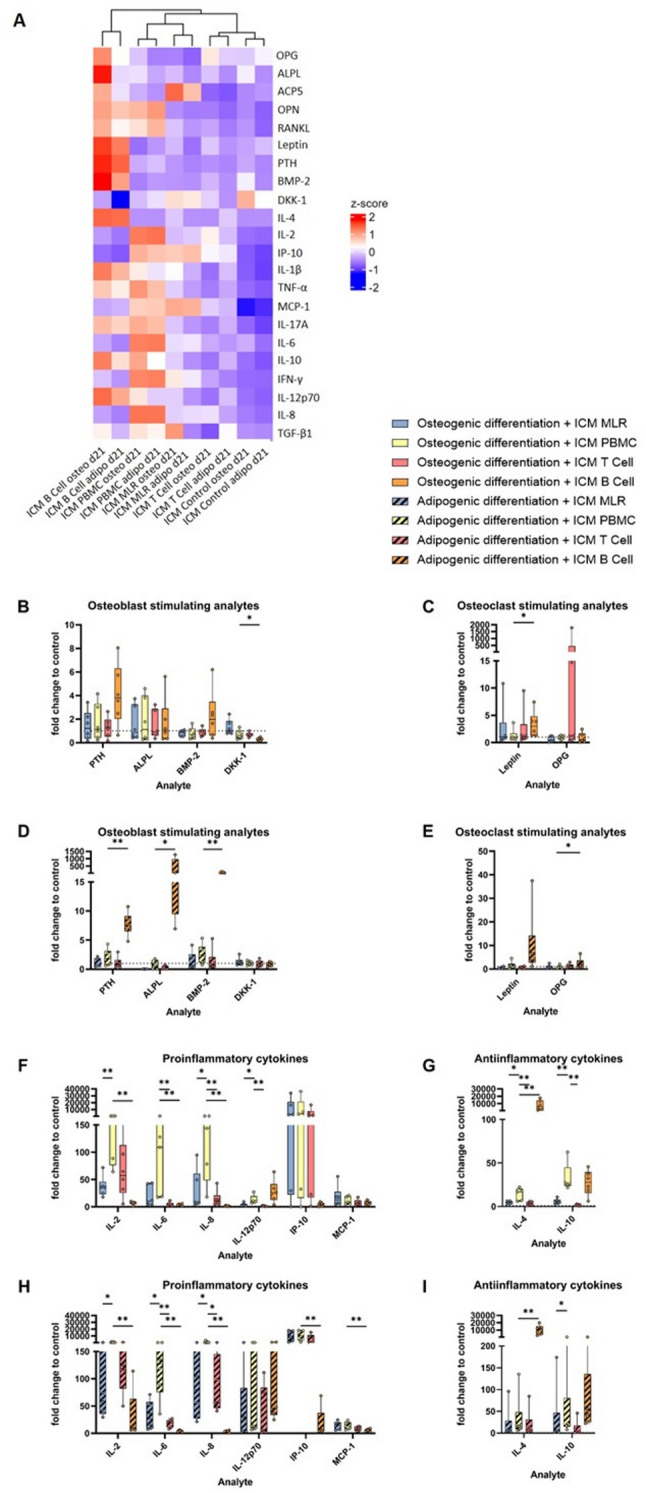
Data of multiplex ELISAs **A**) Results of the multiplex ELISA summarized in a heatmap after performing a z-scoring with the data. **B**-**I** Graphs of selected pro-osteoblastic, pro-osteoclastic, proinflammatory and anti-inflammatory cytokines and proteins after normalization to the control. OM, osteo = osteogenic differentiation, AM, adipo = adipogenic differentiation, ICM MLR = mixed lymphocyte reaction-immune-conditioned medium, ICM PBMC = activated peripheral blood mononuclear cell-immune-conditioned medium, ICM T Cell = activated T Cell-immune-conditioned medium, ICM B Cell = activated B Cell-immune-conditioned medium

MSC culture media were collected at different time points during osteogenic and adipogenic differentiation and the media of day 21 were analyzed via multiplex ELISA. The most evident disparities manifested at the culmination of the differentiation process following a 21-day period (Figure S3). Consequently, the present analysis will concentrate on the data collected on day 21. The heatmap shows that analytes related to immune system modulation were predominantly elevated after MSC stimulation with PBMC-immune-conditioned medium. However, T cell-immune-conditioned medium did not increase the levels of cytokines involved in immune system modulation or in bone metabolism. Additionally, MLR-immune-conditioned medium led to increased amounts of cytokines modulating the immune system. In contrast, analytes involved in bone metabolism increased after MSC stimulation with B cell- and immune-conditioned media (Fig. 6A). Compared with unconditioned control medium, MLR-conditioned medium increased the secretion of the proinflammatory cytokines IL-2, IL-6, IL-8, and IL-12p70 in differentiating MSCs and induced lower secretion of anti-inflammatory cytokines during osteogenic and adipogenic differentiation. The PBMC-immune-conditioned medium also induced increased secretion of proinflammatory cytokines during differentiation, and the secretion of the anti-inflammatory cytokine IL-10 was also induced. The same secretion pattern as that of the MLR-immune-conditioned medium was induced through stimulation with T cell-immune-conditioned medium. Compared with PBMC-immune-conditioned medium, B cell-immune-conditioned media reduced the secretion of most proinflammatory cytokines, with the exception of IL12p70. In addition, B cell-immune-conditioned medium either increased anti-inflammatory cytokine levels (IL-4) or resulted in levels comparable to those observed after stimulation with PBMC-immune-conditioned medium. All these changes were detectable during both osteogenic and adipogenic differentiation, although the most pronounced differences occurred during adipogenic differentiation (Fig. 6B-I).

### Influence of immune-conditioned media on the gene expression of differentiating MSCs

**Fig. 7:**
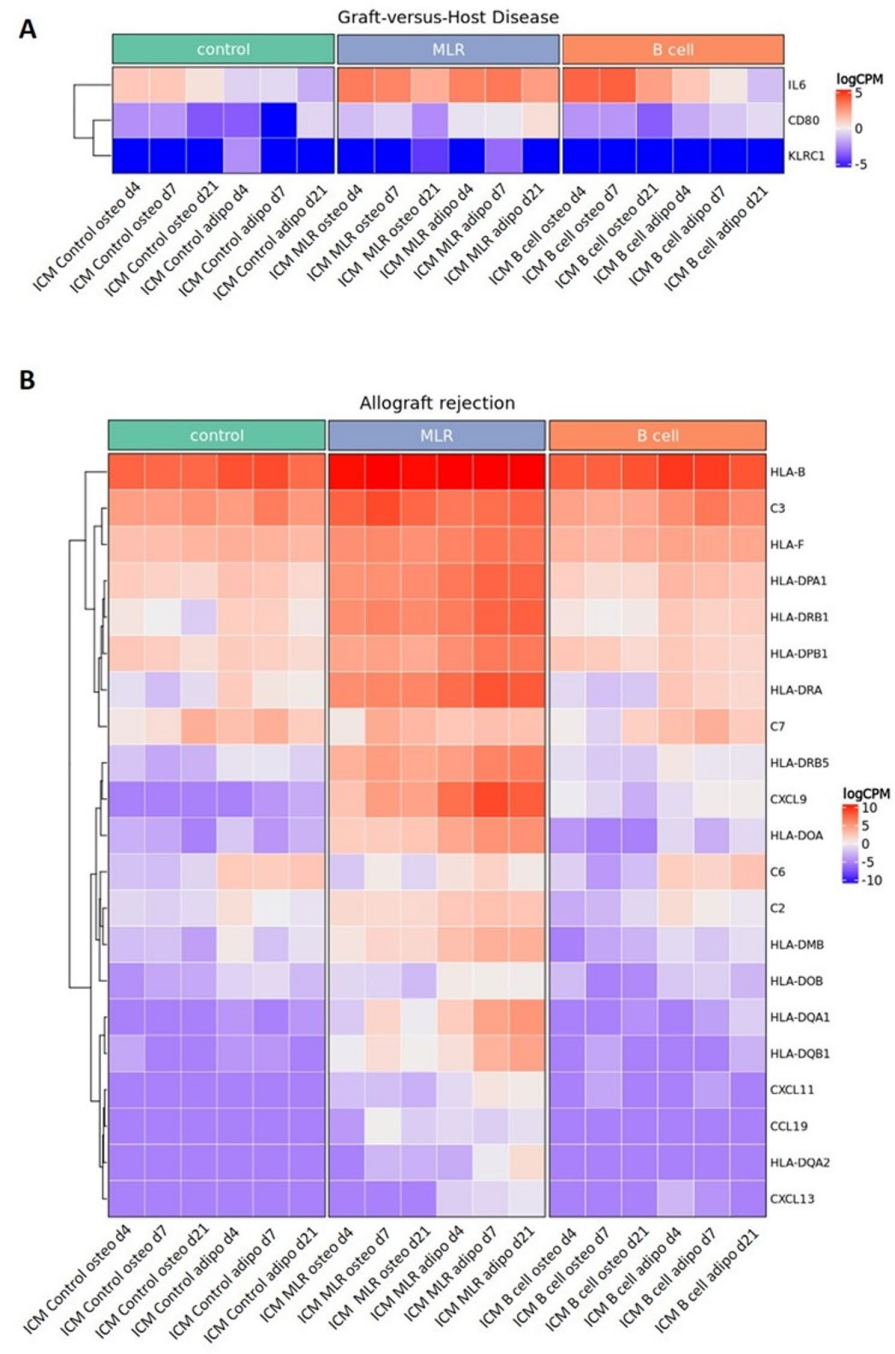
Selected RNA sequencing data. **A** RNA sequencing data of graft-versus-host disease-related genes in differentiated MSCs stimulated with different immune-conditioned media. **B** RNA sequencing data of allograft rejection-related genes in differentiated MSCs stimulated with different immune-conditioned media. OM, osteo = osteogenic differentiation, AM, adipo = adipogenic differentiation, ICM Control = medium control, ICM MLR = mixed lymphocyte reaction immune-conditioned medium, ICM PBMC = activated peripheral blood mononuclear cell immune-conditioned medium, ICM T Cell = activated T cell-immune-conditioned medium, ICM B Cell = activated B cell immune-conditioned medium

MSCs underwent osteogenic and adipogenic differentiation when subjected to immune-conditioned medium. To gain deeper insights into their transcriptional responses to specific immune cell-derived stimuli, RNA sequencing was performed on differentiated MSCs that were unstimulated or stimulated with MLR-immune-conditioned medium or B cell-immune-conditioned medium. The decision to sequence cells from these conditions was made subsequent to the observation that these conditions exhibited the most significant alterations in cell differentiation behavior. RNA sequencing of cells differentiated from MSCs revealed that the different immune-conditioned media induced distinct gene expression profiles the most prominent genes were selected for the heatmaps (Fig. 7A-B). In Fig. 7A we determined the genes that have been documented in the literature as being significantly influenced during GVHD. It should be noted that the three genes under consideration do not represent the entire list of genes implicated in GVHD. In order to maintain focus on the salient aspects of the study, a decision was made to prioritize genes that exhibited significant disparities in gene expression levels following stimulation. MSCs stimulated with MLR immune-conditioned medium induced the upregulation of the IL-6 gene in differentiating MSCs, along with various subgroups of human leukocyte antigens (HLA-B, HLA-F, HLA-DPA1, HLA-DRB1, HLA-DPB1, HLA-DRA, HLA-DRB5, HLA-DOA, HLA-DMB, HLA-DQA1, HLA-DQB1, and HLA-DQA2), proteins of the complement system (C2, C3, C6, and C7), CXC ligands (CXCL9, CXCL11, and CXCL13), and CC ligand 19 (CCL19). Elevated expression of genes encoding HLA subgroups and the complement system has been associated with graft-versus-host disease and allograft rejection [27, 28]. In contrast, B cell-immune-conditioned medium resulted in increased gene expression of interleukin 6 (IL-6) during osteogenic differentiation compared with the control, which did not receive any immune stimulus. This finding suggests that the B cell secretome is less likely to increase the expression of genes involved in antigen presentation during MSC differentiation and, consequently, immune cell activation.

**Fig. 8:**
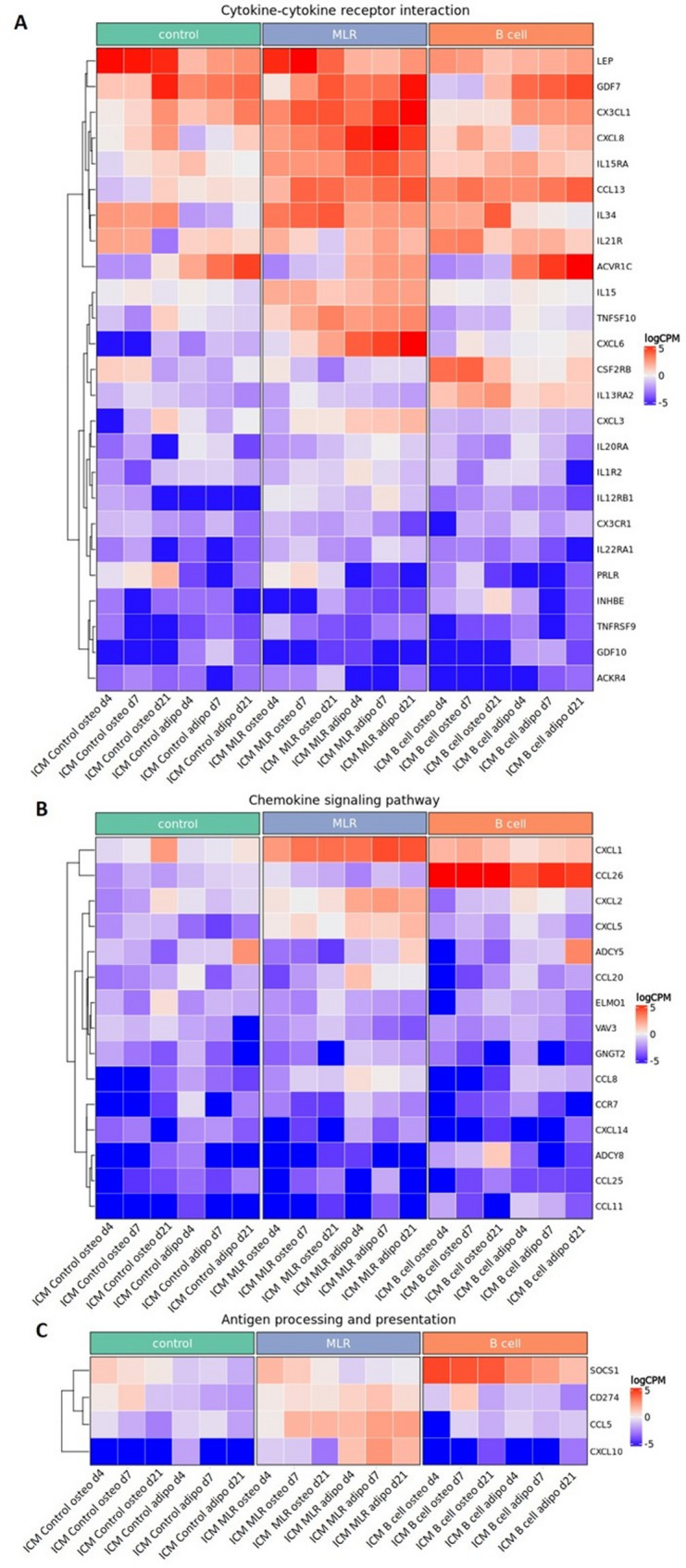
RNA sequencing data. Data of selected genes related to **A**) cytokine‒cytokine receptor interactions, **B**) chemokine signaling pathways and **C**) antigen processing and presentation. Osteo = osteogenic differentiation, Adipo = adipogenic differentiation, ICM Control = medium control, ICM MLR = mixed lymphocyte reaction-immune-conditioned medium, ICM PBMC = activated peripheral blood mononuclear cell immune-conditioned medium, ICM T cell = activated T cell-immune-conditioned medium, ICM B cell = activated B cell-immune-conditioned medium

In Fig. 8, the expression of various genes is shown that are susceptible to modulation in response to immune system activation. A decision was made to divide the genes into three sections. The first section (Fig. 8A) contains genes with more descriptions during cytokine-cytokine interaction. The second section (Fig. 8B) concludes the genes that are more described during chemokine signaling. The third section (Fig. 8C) contains genes that are more described to be influenced during antigen processing and presentation. This is not a comprehensive list of all genes implicated in these processes. However, in order to gain a more comprehensive understanding of the subject, the genes that exhibited the highest differential expression in our studies were extracted. Compared with B cell-immune-conditioned medium, MLR-immune-conditioned medium increased the number of RNA copies of a greater number of genes involved in cytokine‒cytokine receptor interactions (Fig. 8A). Stimulation with MLR-immune-conditioned medium increased the RNA levels of CXCL1, CXCL2 and CXCL5, which are part of the chemokine signaling pathway. CCL20 also increased in differentiating MSCs after stimulation with MLR-immune-conditioned medium but only during adipocytic differentiation. The increased expression of genes involved in cytokine‒cytokine receptor interactions and antigen processing and presentation following stimulation with MLR-immune-conditioned medium suggests that the MLR creates a proinflammatory milieu during the differentiation of MSCs. In contrast, MSC stimulation with B cell-immune-conditioned medium slightly increased CXCL1 expression and elevated CCL26 levels compared with those in control medium (Fig. 8B). The RNA levels of genes involved in antigen processing and presentation were increased in differentiating MSCs following stimulation with B cell-immune-conditioned medium (SOCS1), whereas the CD274, CCL5 and CXCL10 levels were increased after stimulation with MLR-immune-conditioned medium (Fig. 8C).

## Discussion

Bone regeneration following destructive events such as fractures, myeloablative therapies, or bone marrow transplantation is orchestrated by well-regulated interactions between immune cells, including various T cell subsets and the mesenchymal cell lineage. Even slight imbalances or the absence of specific cell types can have a major effect on the overall regenerative capacity. Several studies have investigated the distribution of cells in bone during regeneration, the duration of cellular reconstitution after transplantation, and late side effects that may arise if regeneration fails [3–5]. To our knowledge, however, little is known about which specific cells or combinations of cells of the adaptive immune system influence the differentiation capacity of MSCs. Given the limited knowledge in this area, we conducted a series of experiments to determine the extent to which various subsets of the adaptive immune system influence the osteogenic and adipogenic differentiation capacity of MSCs, as these are key processes for bone health. Our findings suggest that activated B cells significantly affect the matrix production of differentiating MSCs in cell culture, with the potential to promote a positive regenerative response.

The prerequisite for obtaining these results is the use of physiological immune stimuli. These stimuli were chosen for their ability to effectively and specifically induce the production of different immune-conditioned media. We prepared immune-conditioned media that exhibited cytokine levels within the physiological activation range of the targeted immune cells [29–31], which are also likely to be within the physiological range encountered by MSCs within the bone environment. As a result, stimulation of differentiating MSCs with specific immune-conditioned media induced responses that mirrored the physiological behavior of MSCs during bone regeneration, a process initiated by activated immune cells.

To elicit a mixed lymphocyte reaction, blood samples from two donors whose compatibility had not been previously tested were used. Since the probability of finding two compatible donors is low, instead, the medium was examined for markers indicative of donor incompatibility. These markers include components of the complement system. We were able to show that cytokine secretion during the mixed lymphocyte reaction indeed showed donor incompatibility and was sufficient to induce increased expression of genes associated with allograft rejection and graft-versus-host disease in differentiating MSCs. An increase in CXCL9 expression is an indicator of allograft rejection, as elevated CXCL9 levels have been directly correlated with the incidence of allograft rejection in patients following renal, lung, and liver transplantation [32–35]. The upregulation of genes encoding complement components such as C3, C7, C2 and C6 further supports the hypothesis that allograft rejection pathways are activated. After transplantation, alloreactive T cells are induced, whereas regulatory T cells are suppressed [36]. The activation of the complement cascade during this process has been implicated in allograft injury. Moreover, the complement cascade induces allograft injury through ischemic reperfusion, ultimately leading to allograft dysfunction [37, 38]. The third group of genes upregulated in differentiating MSCs by MLR-immune-conditioned medium comprises various HLA subtypes. The role of HLA in antigen processing and presentation is critical for the activation of alloreactive T cells. Disruption of HLA alleles has been shown to increase the immune compatibility of cells, indicating that increased HLA expression promotes stronger immune cell responses [27, 28]. Thus, even though the incompatibility of the donors used for the MLR was not tested, the data proved that the immune-immune-conditioned medium reflected the signaling components of the assumed incompatibility.

The activation of PBMCs and T cells via anti-CD3 and anti-CD28 antibodies results in immune-conditioned media activated via a well-established protocol. However, the T cell stimulation resulted in the production of an immune-conditioned medium, characterized by low levels of cytokine secretion. It is conceivable that the T cells of the donor had become exhausted due to a preliminary infection. Consequently, the T cell immune-conditioned medium may not have been as effective as anticipated. Even so, preliminary research into the existing literature on the subject suggested a negative impact of T cell activation on the osteogenic differentiation of MSCs [39, 40]. Despite the minimal T cell activation observed, the anticipated adverse effect was evident in our study, thereby substantiating the activation as efficacious.

The utilization of anti-IgM and anti-IL-4 antibodies for the purpose of B cell activation was subsequently selected for comprehensive literature research and activation testing. The subsequent pattern demonstrated that B cell-immune-conditioned medium did not increase the gene expression levels of IL-6 in adipogenic differentiated MSCs. Furthermore, the complement factors C2, C3, and C7 and the HLA subtypes with augmented gene expression levels subsequent to MLR-immune-conditioned medium stimulation presented expression levels that were congruent with those observed in the control group.

Different types of immune cell stimulation of adaptive immune system components result in specific immune-conditioned media, which are then applied during the differentiation process of MSCs. Stimulation of differentiating MSCs with B cell-immune-conditioned medium enhanced mineralized matrix deposition, whereas stimulation with other immune-conditioned media did not significantly affect the osteogenic differentiation capacity. In agreement with this observation, stimulation with MLR-, PBMC-, and T cell-conditioned media resulted in lower collagen production ratios during the osteogenic differentiation of MSCs. It has been reported that T cells can increase osteoclastogenesis and therefore enhance bone resorption [41]. In contrast, Qiu et al. also hypothesized that B cells may influence osteoblast differentiation but suggested that further studies are needed to clarify the nature of this effect [42]. We and others have investigated the distribution of B cells during fracture healing and showed that both their number and localization change over the course of the healing process. In the initial phase of fracture healing, there is an increase in the number of B cells in the vicinity of the fracture gap, indicating that these cells are highly involved in the process of bone homeostasis. The B cell number subsequently decreases, followed by a renewed increase during the late phase of fracture healing, when the B cells relocate into the fracture gap [4, 43]. These findings support the results of this study that B cells are essential for bone health when the balance of the cellular composition is disturbed. The beneficial milieu for adipocytic differentiation, induced by the greater number of activated B cells, helps to increase the energy supply during bone injury.

Furthermore, we observed a decrease in adipocyte formation when MSCs were stimulated with MLR-, PBMC-, and T cell-immune-conditioned media. Despite the prevailing view that adipocytes negatively impact bone formation, a growing body of research challenges this assumption by suggesting a potentially beneficial role for these cells [44, 45]. The results we obtained indicate that adipocytes have a beneficial effect, which appears to be orchestrated by B cells rather than T cells. Notably, however, T cells predominate in a mixed lymphocyte response and during the activation of PBMCs. Zhou et al. reported that the cytopenia induced by irradiation was reversed when the bone marrow contained a greater proportion of adipocytes [46]. Additionally, Zhou et al. and Burkhardt et al. reported that adipocytes release factors that induce HSC reconstitution, which are normally produced by HSC niche components [46, 47]. In conclusion, our results suggest that a greater number of adipocytes could bridge the delay in the recovery of HSC niches by facilitating the secretion of cytokines, that promote HSC reconstitution.

All immune-conditioned media used in this study contained elevated levels of proinflammatory cytokines, as shown by analysis of IL-2, IL-6, IL-8, IL-1β, IFNγ, TNF-α and TGF-β, which are key regulators of the immune response. However, only the B cell-immune-conditioned medium exhibited a signaling profile that included increased levels of anti-inflammatory cytokines. IL-4 was utilized for the activation of B cells. However, the measured amount of IL-4 was found to exceed the added amount. In addition, the half-life of IL-4 in culture experiments was examined and reported to be less than 25 h. Furthermore, IL-4, in the absence of glycosilation, exhibits a half-life that ranges from minutes to a few hours [48]. IL-4 added here was not glycosilated and the immune-conditioned medium was harvested 48 h after the addition of IL-4. An increased release of proinflammatory cytokines, especially IL-6, has been shown to impair bone regeneration and promote bone loss by inducing senescence in stem cells [49]. Furthermore, IL-8 stimulation of MSCs led to decreased formation of bone nodules [50]. Anti-inflammatory cytokines, such as IL-10, have been shown to promote the polarization of M1 macrophages to M2 macrophages and decrease the production of proinflammatory cytokines. M2 macrophages are beneficial for regenerative processes [51, 52], which are supported by the cytokines secreted by B cells [4, 53]. Moreover, increased IL-4 levels have been utilized as a therapeutic approach against inflammaging, promoting osteoblast differentiation over proliferation and thereby enhancing bone formation [53–55].

Furthermore, we tested the effects of immune-conditioned media on the secretion of key regulators of bone metabolism. The MLR-immune-conditioned medium resulted in elevated levels of ACP5, OPN, DKK-1 and IL-6. Furthermore, the activation of PBMCs resulted in increased secretion of ACP5, OPN, DKK-1, IL-6 and TNF-α. The B cell-immune-conditioned medium increased the levels of osteoblast- and osteoclast-stimulating proteins and cytokines, including PTH, ACP5, RANKL, OPN, ALPL, BMP-2, DKK-1, leptin, OPG, IL-6, TNF-α and IL-1β. The importance of B cells and T cells in maintaining bone homeostasis and contributing to various stages of fracture healing is well supported by numerous studies demonstrating the interactions between their secreted factors, OPG and RANKL, and osteoblasts and osteoclasts. In addition, evidence has emerged indicating that T cells, when cultivated in isolation from B cells, tend to induce bone loss through the secretion of PTH [41, 56–58]. Our data demonstrated that elevated levels of both OPG and RANKL secretion were observed exclusively in the B cell-immune-conditioned medium. This finding indicates that activated B cells may play a role in fostering a pro-osteoclastic environment within the bone tissue. Treatment with B cell-immune-conditioned medium also increased ALPL and Leptin secretion during MSC differentiation. Research indicates that elevated ALPL levels prevent MSC senescence and promote MSC differentiation [59]. In addition, ALPL positively influences the proangiogenic capacity of MSCs [60]. In comparison with the control group, the secretion of ALPL is notably augmented following B cell-immune-conditioned medium stimulation during the process of adipogenic differentiation. This finding suggests that B cell-immune-conditioned medium may play a role in the osteogenic support of bone marrow adipocytes. In recent years, there has been an uptick in research interest in bone marrow adipocytes. This interest stems from two key findings. First, adipocytes have been shown to provide energy during the initial phase of bone healing. Second, under specific conditions, bone marrow adipocytes have the capacity to transdifferentiate into osteoblasts [8, 61]. MSCs expressing the leptin receptor serve as the primary progenitors for both bone cells and adipocytes. Higher levels of leptin have been demonstrated to increase the perimeter, number, and activity of osteoblasts. Additionally, higher levels of leptin have been shown to augment the mineralizing perimeter and overall bone formation rate [62–64]. In summary, this study demonstrated that stimulation with B cell-immune-conditioned medium increased the secretion of factors that promote bone turnover. In contrast, T cell-immune-conditioned medium has been shown to induce a bone resorption milieu through elevated PTH secretion.

RNA sequencing revealed that stimulation of MSCs with MLR-immune-conditioned medium led to the strongest upregulation of genes involved in cytokine‒cytokine receptor interactions, allograft rejection, antigen processing and presentation and graft‒verus-host disease pathways. MLR-conditioned medium notably increased the expression of chemokines, with CXCL1, CXCL2 and CXCL5 showing the most pronounced upregulation. In contrast, B cell-immune-conditioned medium led to an increase in CCL26 during MSC differentiation. Cytokines such as CXCL1, CXCL2 and CXCL5 are associated with neutrophil recruitment and the induction of a proinflammatory milieu. In addition, CXCL1 has been implicated in the development of osteoporosis and rheumatoid arthritis, whereas increased CXCL2 expression has been linked to the premature senescence of MSCs [65–67]. In contrast to CXCL1, CXCL2 and CXCL5, which attract neutrophils, CCL26 functions primarily as a chemoattractant for eosinophils [68]. On the basis of current knowledge, neutrophils have been shown to activate osteoclasts and contribute to bone loss under inflammatory conditions, whereas eosinophils do not appear to exert detrimental effects on bone. Eosinophils have been described as beneficial for the survival of plasma cells in the bone, thereby helping to maintain immunological memory [69]. In summary, the administration of MLR-immune-conditioned medium resulted in diminished mineralized matrix production. Consequently, the immune-conditioned medium exhibited cytokine levels, which adversely impacted the differentiation capacity of MSCs. The findings reported here are consistent with the adverse effects observed in stem cell transplants in the context of graft-versus-host disease. The results from stimulating differentiating MSCs with B cell- and T cell-immune-conditioned media further support these findings. T cell-immune-conditioned medium exerts a predominantly negative effect on MSC differentiation, whereas B cell-immune-conditioned medium induces this process and could therefore be considered a factor in bone health.

This study is not without its limitations. Notwithstanding the utilization of MSCs from six donors, the immune cells employed in the production of the immune-conditioned media were derived from a single donor. Given the potential for immune cells from different donors to exhibit divergent activation processes, it is conceivable that immune cells from alternative donors could elicit distinct cytokine levels in the immune-conditioned media. In the context of single-donor isolation for the purpose of obtaining PBMCs, it is imperative to acknowledge the potential donor-dependent nature of the resulting immune-conditioned media. While the influence of the donor is acknowledged, it is superseded by the significance of the activation protocol in determining the ICM’s characteristics. In addition, the process of immune cell activation is contingent upon the isolation, cultivation, and current health status of the donor. Furthermore, the composition of the ICM is contingent upon temporal and energetic factors. All of these variables will exert an influence on the cytokine pattern. In the present study, our objective was not to elucidate differences in signaling. Nevertheless, we think it is not a limitation using one immune cell donor, as the composition of the conditioned medium is important for the cellular reaction – and the cytokine pattern is well analyzed and reported in the manuscript. Including different conditioned media and different MSC donor (and we put more weight on including different MSC donor) would increase the variability to an unmanageable level. The other solution would have been to combine conditioned medium from different immune cell donors – but in that case we nevertheless would have done the experiments with only one cytokine pattern. The analyses of the conditioned medium did show a pattern that was to be expected for the specific immune subset – and thus was deemed representable. A notable constraint in our study is the observation that the activation of T cells resulted in diminished levels of cytokine secretion compared to our initial expectations. Despite the diminished cytokine levels, the medium exhibited discrepancies in the differentiation capacity of MSCs following stimulation and therefore is shown in this study. Furthermore, the study investigated the impact of adaptive immune cell types on the differentiation potential of MSCs. It is acknowledged that innate immune cell influence on inflammatory processes during bone healing has been previously discussed in relation to PBMC-immune-conditioned media. However, the present study centered on the influence of adaptive immunity. Therefore, it must be acknowledged that PBMC isolates are comprised of up to 10% myeloid cells. Myeloid cells, comprising monocytes, have the capacity to differentiate into macrophages. Macrophages are recognized for their pivotal role in facilitating bone healing, a process in which they play a pivotal regulatory role. These cells secrete inflammatory cytokines and growth factors that can induce osteoblastic differentiation of MSCs. Monocytes have also been demonstrated to possess the capacity to induce the proliferation of MSCs when utilized in elevated concentrations [70, 71]. However, given that no cytokines were added to induce monocyte differentiation into macrophages, and given that no induction of osteoblastic differentiation was observed following stimulation of MSCs with PBMC- or MLR-immune-conditioned medium compared to T cell-immune-conditioned medium, the influence of myeloid cells in the utilized isolates was not a focal point of the study. Moreover, the influence of innate immunity has been the subject of extensive research over the years. The objective of this study was to broaden the existing body of knowledge concerning the impact of the adaptive immune system on bone regeneration. To this end, the adaptive immune system was examined in detail.

While the present study does not address experiments involving the neutralization or depletion of B cells in animals, it nevertheless underscores the importance of B cells for maintaining bone homeostasis. This assertion is further substantiated by the findings of our preceding studies. As we previously demonstrated, B cells are present in the immediate vicinity of the fracture gap during the process of fracture healing [4]. In addition, mouse experiments have demonstrated that the process of bone marrow regeneration following stem cell transplantation is prolonged and that alterations to the bone structure persist as long as there are no B cells or only a greatly reduced number of B cells present in the bone marrow [3]. In light of the findings, a subsequent investigation was deemed necessary to further explore the impact of immune cells on the differentiation of MSCs. In addition, activated B cells have been shown to secrete both pro- and anti-inflammatory cytokines, thereby creating a milieu that is essential for optimal bone regeneration. Furthermore, B cells secrete pro-osteoclastic and pro-osteoblastic cytokines and proteins, thereby facilitating bone remodeling. The present study demonstrated that stimulating differentiating MSCs with B cell-immune-conditioned medium has a positive effect on matrix mineralization and adipocyte formation. The effects of B cells are clearly important for the maintenance of bone homeostasis. This is particularly noteworthy given that T cell-conditioned medium did not elicit these effects; instead, it impaired the MSC differentiation capacity due to the predominance of a proinflammatory cytokine milieu.

While the interdependency of cell subsets during bone health has been shown to be important, we have demonstrated the pivotal role of B cells in maintaining a balanced milieu for bone homeostasis and the underlying mechanisms that govern their presence in bone tissue. In the context of pathological bone regeneration events and the development of new therapies, with the aim of destroying the balance of cells in the immune system or depleting a whole subset of immune cells, it is important to acknowledge the importance of B cells for bone health, with the goal of preserving a balanced ratio of B cells and T cells.

## Supplementary Information

Supplementary Material 1.

## Data Availability

All data supporting the findings of this study are available within the paper.

## Abbreviations

ACP-5: Acid Phosphatase 5
ALPL: Alkaline Phosphatase
AM: Adipogenic Medium
BSA: Bovine Serum Albumin
BMP-2: Bone Morphogenetic Protein 2
CCL: CC Motif Chemokine Ligand
CXCL: C-X-C Motif Chemokine Ligand
DMEM: Dulbecco’s Modified Eagle Medium
DKK-1: Dickkopf-1
DNA: Desoxyribonucleic acid
DNAse: Desoxyribonuclease
EA: Ethical Approval
EDTA: Ethylenediaminetetraacetat
ELISA: Enzyme-Linked Immunosorbent Assay
FABP4: Fatty Acid Binding Protein 4
FBS: Fetal Bovine Serum
FCS: Fetal Calf Serum
HLA: Human Leukocyte Antigen
HRP: Horseradish Peroxidase
HSC: Hematopoietic Stem Cell
HSCT: Hematopoietic Stem Cell Transplantation
ICM: Immune-Conditioned Medium
IFNγ: Interferon Gamma
IgM: Immunglobulin M
IL: Interleukin
IP-10: Interferon Gamma-Induced Protein 10 (CXCL10)
MCP-1: Monocyte Chemoattractant Protein 1 (CCL2)
MLR: Mixed Lymphocyte Reaction
MSC: Mesenchymal Stromal Cell
OM: Osteogenic Medium
OPG: Osteoprotegerin
OPN: Osteopontin
PBS: Phosphate Buffered Saline
PBMC: Peripheral Blood Mononuclear Cell
PTH: Parathyroid Hormone
RNA: Ribonucleic Acid
RPMI Medium: Rosswell Park Memorial Institute Medium
SOCS1: Suppressor of Cytokine Signaling 1
TGF-β: Transforming Growth Factor Beta
TMB: Tetramethylbenzidine
TNF-α: Tumor Necrosis Factor Alpha

## References

[1] Bonelli M, Brunelli S, Schildberg FA, Guder C, Gravius S, Burger C et al. Osteoimmunology: A Current Update of the Interplay Between Bone and the Immune System. Front Immunol | www.frontiersin.org. 2020 [cited 2024 Mar 11];1:58. Available from: https://www.frontiersin.org. 10.3389/fimmu.2020.00058 PMC7004969 32082321

[2] Tsukasaki M, Takayanagi H. Osteoimmunology: evolving concepts in bone–immune interactions in health and disease. [cited 2024 Mar 11]; Available from: https://doi.org/10.1038/. 10.1038/s41577-019-0178-8 31186549

[3] Schwarz CS, Bucher CH, Schlundt C, Mertlitz S, Riesner K, Kalupa M et al. Spatio-Temporal Bone Remodeling after Hematopoietic Stem Cell Transplantation. Int J Mol Sci. 2020 Jan 1 [cited 2024 Mar 13];22(1):1–14. Available from: https://pubmed.ncbi.nlm.nih.gov/33383915/. 10.3390/ijms22010267 PMC7795370 33383915

[4] Schlundt C, Saß RA, Bucher CH, Bartosch S, Hauser AE, Volk HD et al. Complex Spatio-Temporal Interplay of Distinct Immune and Bone Cell Subsets during Bone Fracture Healing. Cells. 2023 Jan 1 [cited 2024 Mar 13];13(1). Available from: https://pubmed.ncbi.nlm.nih.gov/38201244/. 10.3390/cells13010040 PMC10777943 38201244

[5] Bucher CH, Lei H, Duda GN, Schmidt-Bleek HDV, Bucher K, Lei CH et al. H,. The Role of Immune Reactivity in Bone Regeneration. Adv Tech Bone Regen. 2016 Aug 31 [cited 2023 Aug 10]; Available from: https://www.intechopen.com/chapters/50169.

[6] Vonkeman HE, Fernandes RW, Van der Palen J, Van Roon EN, Van de Laar MAFJ. Differential expression of RANK, RANK-L, and osteoprotegerin by synovial fluid neutrophils from patients with rheumatoid arthritis and by healthy human blood neutrophils. Arthritis Res Ther. 2007 May 23 [cited 2026 Jan 28];9(2):R25. Available from: https://pmc.ncbi.nlm.nih.gov/articles/PMC1906801/. 10.1186/ar2137 PMC1906801 17341304

[7] Sato K, Suematsu A, Okamoto K, Yamaguchi A, Morishita Y, Kadono Y et al. Th17 functions as an osteoclastogenic helper T cell subset that links T cell activation and bone destruction. J Exp Med. 2006 Nov [cited 2026 Jan 28];203(12):2673. Available from: https://pmc.ncbi.nlm.nih.gov/articles/PMC2118166/. 10.1084/jem.20061775 PMC2118166 17088434

[8] Burkhardt LM, Bucher CH, Löffler J, Rinne C, Duda GN, Geissler S, et al. The benefits of adipocyte metabolism in bone health and regeneration. Front Cell Dev Biol. 2023;11:147. 10.3389/fcell.2023.1104709 PMC9988968 36895792

[9] Scheller EL, Doucette CR, Learman BS, Cawthorn WP, Khandaker S, Schell B, et al. Region-specific variation in the properties of skeletal adipocytes reveals regulated and constitutive marrow adipose tissues. Nat Commun. 2015;6(1):1–15. 10.1038/ncomms8808 PMC4530473 26245716

[10] Knight MN, Hankenson KD. Mesenchymal Stem Cells in Bone Regeneration. Adv Wound Care. 2013 Jul [cited 2025 Nov 3];2(6):306. Available from: https://pmc.ncbi.nlm.nih.gov/articles/PMC3842877/. 10.1089/wound.2012.0420 PMC3842877 24527352

[11] Marnitz S, Zich A, Martus P, Budach V, Jahn U, Neumann O, et al. Long-term results of total body irradiation in adults with acute lymphoblastic leukemia. Strahlentherapie und Onkol. 2014;190(5):453–8. 10.1007/s00066-014-0607-3 24595415

[12] Majhail NS, Ness KK, Burns LJ, Sun CL, Carter A, Francisco L, et al. Late Effects in Survivors of Hodgkin and Non-Hodgkin Lymphoma Treated with Autologous Hematopoietic Cell Transplantation: A Report from the Bone Marrow Transplant Survivor Study. Biol Blood Marrow Transpl. 2007;13(10):1153–9. 10.1016/j.bbmt.2007.06.003 PMC2083636 17889351

[13] Savani BN, Donohue T, Kozanas E, Shenoy A, Singh AK, Childs RW, et al. Increased Risk of Bone Loss without Fracture Risk in Long-Term Survivors after Allogeneic Stem Cell Transplantation. Biol Blood Marrow Transpl. 2007;13(5):517–20. 10.1016/j.bbmt.2007.01.085 17448910

[14] Yu VWC, Scadden DT. Hematopoietic Stem Cell and Its Bone Marrow Niche. Current Topics in Developmental Biology. Academic Press Inc. 2016:118:21–44. 10.1016/bs.ctdb.2016.01.009. 10.1016/bs.ctdb.2016.01.009 PMC6854531 27137653

[15] Ara T, Tokoyoda K, Sugiyama T, Egawa T, Kawabata K, Nagasawa T. Long-term hematopoietic stem cells require stromal cell-derived factor-1 for colonizing bone marrow during ontogeny. Immunity. 2003;19(2):257–67. 12932359 10.1016/s1074-7613(03)00201-2

[16] Zhang J, Niu C, Ye L, Huang H, He X, Tong WG, et al. Identification of the haematopoietic stem cell niche and control of the niche size. Nature. 2003;425(6960):836–41. 14574412 10.1038/nature02041

[17] Diaz MF, Horton P, lina D, Dumbali SP, Kumar A, Livingston M, Skibber MA et al. Bone marrow stromal cell therapy improves survival after radiation injury but does not restore endogenous hematopoiesis. Sci Rep. 2020 Dec 1 [cited 2022 Jul 5];10(1):22211. Available from: https:///pmc/articles/PMC7747726/. 10.1038/s41598-020-79278-y PMC7747726 33335275

[18] Fistonich C, Zehentmeier S, Bednarski JJ, Miao R, Schjerven H, Sleckman BP, et al. Cell circuits between B cell progenitors and IL-7 + mesenchymal progenitor cells control B cell development. J Exp Med. 2018;215(10):2586–99. 30158115 10.1084/jem.20180778 PMC6170173

[19] Yu VWC, Saez B, Cook C, Lotinun S, Pardo-Saganta A, Wang YH, et al. Specific bone cells produce DLL4 to generate thymus-seeding progenitors from bone marrow. J Exp Med. 2015;212(5):759–74. 25918341 10.1084/jem.20141843 PMC4419348

[20] Search ClinicalTrials. gov for: Other terms: B cell depletion | Card Results | ClinicalTrials.gov. [cited 2025 Sep 7]. Available from: https://clinicaltrials.gov/search?term=B cell depletion.

[21] Abeles I, Palma C, Meednu N, Payne AS, Looney RJ, Anolik JH. B Cell-Directed Therapy in Autoimmunity. 2026 [cited 2026 Feb 11];44:25. Available from: 10.1146/annurev-immunol-083122- 10.1146/annurev-immunol-083122-044829 38011889

[22] Lee DSW, Rojas OL, Gommerman JL. B cell depletion therapies in autoimmune disease: advances and mechanistic insights. Nat Rev Drug Discov. 2020 Mar 1 [cited 2026 Feb 11];20(3):179. Available from: https://pmc.ncbi.nlm.nih.gov/articles/PMC7737718/. 10.1038/s41573-020-00092-2 PMC7737718 33324003

[23] Autissier P, Soulas C, Burdo TH, Williams KC. Evaluation of a 12-Color Flow Cytometry Panel to Study Lymphocyte, Monocyte, and Dendritic Cell Subsets in Humans. 2010 [cited 2026 Jul 4]; Available from: https://onlinelibrary.wiley.com/doi/10.1002/cyto.a.20859. 10.1002/cyto.a.20859 PMC11742174 20099249

[24] Kleiveland C, Kleiveland C. Peripheral Blood Mononuclear Cells. Impact Food Bioact Heal Vitr Ex Vivo Model. 2015 Jan 1 [cited 2026 Mar 14];161–7. Available from: https://www.ncbi.nlm.nih.gov/books/NBK500157/.

[25] Reinke S, Dienelt A, Blankenstein A, Duda GN, Geissler S. Qualifying stem cell sources: How to overcome potential pitfalls in regenerative medicine? J Tissue Eng Regen Med. 2016;10(1):3–10. 24919850 10.1002/term.1923

[26] Ode A, Duda GN, Glaeser JD, Matziolis G, Frauenschuh S, Perka C, et al. Toward biomimetic materials in bone regeneration: Functional behavior of mesenchymal stem cells on a broad spectrum of extracellular matrix components. J Biomed Mater Res - Part A. 2010;95(4):1114–24. 10.1002/jbm.a.32909 20878902

[27] Xu H, Wang B, Ono M, Kagita A, Fujii K, Sasakawa N, et al. Targeted Disruption of HLA Genes via CRISPR-Cas9 Generates iPSCs with Enhanced Immune Compatibility. Cell Stem Cell. 2019;24(4):566–e5787. 30853558 10.1016/j.stem.2019.02.005

[28] Dendrou CA, Petersen J, Rossjohn J, Fugger L. HLA variation and disease. Nat Rev Immunol 2017 185. 2018 Jan 2 [cited 2024 Sep 20];18(5):325–39. Available from: https://www.nature.com/articles/nri.2017.143. 10.1038/nri.2017.143 29292391

[29] Lee HS, Jeong GS. Chrysophanol mitigates T cell activation by regulating the expression of CD40 ligand in activated T cells. Int J Mol Sci. 2020 Sep 1 [cited 2025 May 27];21(17):1–13. Available from: https://pubmed.ncbi.nlm.nih.gov/32854357/. 10.3390/ijms21176122 PMC7504217 32854357

[30] Baccianti F, Masson C, Delecluse S, Li Z, Poirey R, Delecluse HJ. Epstein-Barr virus infectious particles initiate B cell transformation and modulate cytokine response. MBio. 2023 Oct 1 [cited 2025 May 27];14(5). Available from: https://doi.org/10.1128/mbio.01784-23?download=true. 10.1128/mbio.01784-23 PMC10653912 37830871

[31] De Gruijter NM, Jebson B, Rosser EC. Cytokine production by human B cells: role in health and autoimmune disease. Clin Exp Immunol. 2022 Dec 1 [cited 2025 May 27];210(3):253–62. Available from: https://pubmed.ncbi.nlm.nih.gov/36179248/. 10.1093/cei/uxac090 PMC9985175 36179248

[32] Raschzok N, Reutzel-Selke A, Schmuck RB, Morgul MH, Gauger U, Prabowo KA et al. CD44 and CXCL9 serum protein levels predict the risk of clinically significant allograft rejection after liver transplantation. Liver Transplant. 2015 Sep 1 [cited 2024 Sep 19];21(9):1195–207. Available from: https://onlinelibrary.wiley.com/doi/full/10.1002/lt.24164. 10.1002/lt.24164 25950774

[33] Belperio JA, Keane MP, Burdick MD, Lynch JP, Zisman DA, Xue YY et al. Role of CXCL9/CXCR3 Chemokine Biology during Pathogenesis of Acute Lung Allograft Rejection. J Immunol. 2003 Nov 1 [cited 2024 Sep 19];171(9):4844–52. Available from: 10.4049/jimmunol.171.9.4844. 10.4049/jimmunol.171.9.4844 14568964

[34] Shino MY, Todd JL, Neely ML, Kirchner J, Frankel CW, Snyder LD, et al. Plasma CXCL9 and CXCL10 at allograft injury predict chronic lung allograft dysfunction. Am J Transpl. 2022;22(9):2169–79. 10.1111/ajt.17108 PMC9427677 35634722

[35] Ostojic A, Markotic A, Kelava T, Mrzljak A. Association between CXCL9/10 polymorphisms and acute rejection of liver allograft. Medicine (Baltimore). 2019 Feb 1 [cited 2024 Sep 19];98(8). Available from: https:///pmc/articles/PMC6408087/. 10.1097/MD.0000000000014612 PMC6408087 30813187

[36] Sheen JH, Heeger PS. Effects of complement activation on allograft injury. Curr Opin Organ Transplant. 2015 Aug 15 [cited 2024 Sep 19];20(4):468. Available from: https:///pmc/articles/PMC4510836/. 10.1097/MOT.0000000000000216 PMC4510836 26132735

[37] Santarsiero D, Aiello S. The Complement System in Kidney Transplantation. Cells. 2023, Vol 12, Page 791. 2023 Mar 2 [cited 2024 Sep 19];12(5):791. Available from: https://www.mdpi.com/2073-4409/12/5/791/htm. 10.3390/cells12050791 PMC10001167 36899927

[38] Ali HA, Pavlisko EN, Snyder LD, Frank M, Palmer SM. Complement system in lung transplantation. Clin Transplant. 2018 Apr 1 [cited 2024 Sep 19];32(4):e13208. Available from: https://onlinelibrary.wiley.com/doi/full/10.1111/ctr.13208. 10.1111/ctr.13208 29374425

[39] Wu X, Wang W, Meng C, Yang S, Duan D, Xu W et al. Regulation of differentiation in trabecular bone-derived mesenchymal stem cells by T cell activation and inflammation. Oncol Rep. 2013 Nov 1 [cited 2026 Mar 28];30(5):2211–9. Available from: http://www.spandidos-publications.com/10.3892/or.2013.2687/abstract. 10.3892/or.2013.2687 23970332

[40] Grassi F, Cattini L, Gambari L, Manferdini C, Piacentini A, Gabusi E et al. T cell subsets differently regulate osteogenic differentiation of human mesenchymal stromal cells in vitro. J Tissue Eng Regen Med. 2016 Apr 1 [cited 2026 Mar 28];10(4):305–14. Available from: 10.1002/term.1727. 10.1002/term.1727 23653421

[41] Gao Y, Wu X, Terauchi M, Li JY, Grassi F, Galley S, et al. T Cells Potentiate PTH-Induced Cortical Bone Loss through CD40L Signaling. Cell Metab. 2008;8(2):132–45. 18680714 10.1016/j.cmet.2008.07.001 PMC2569843

[42] Qiu X, Liu Y, Shen H, Wang Z, Gong Y, Yang J et al. Single-cell RNA sequencing of human femoral head in vivo. Aging (Albany NY). 2021 Jun 10 [cited 2024 Sep 23];13(11):15595–619. Available from: https://www.aging-us.com/article/203124. 10.18632/aging.203124 PMC8221309 34111027

[43] Zhang H, Wang R, Wang G, Zhang B, Wang C, Li D, et al. Single-Cell RNA Sequencing Reveals B Cells Are Important Regulators in Fracture Healing. Front Endocrinol (Lausanne). 2021;12(November):1–12. 10.3389/fendo.2021.666140 PMC8606664 34819916

[44] Ambrosi TH, Scialdone A, Graja A, Gohlke S, Jank AM, Bocian C, et al. Adipocyte Accumulation in the Bone Marrow during Obesity and Aging Impairs Stem Cell-Based Hematopoietic and Bone Regeneration. Cell Stem Cell. 2017;20(6):771–e7846. 28330582 10.1016/j.stem.2017.02.009 PMC5459794

[45] Woods GN, Ewing SK, Schafer AL, Gudnason V, Sigurdsson S, Lang T et al. Saturated and Unsaturated Bone Marrow Lipids Have Distinct Effects on Bone Density and Fracture Risk in Older Adults. J Bone Miner Res. 2022 Apr 1 [cited 2024 Sep 24];37(4):700–10. Available from: 10.1002/jbmr.4504. 10.1002/jbmr.4504 PMC9018474 35038186

[46] Zhou BO, Yu H, Yue R, Zhao Z, Rios JJ, Naveiras O et al. Bone marrow adipocytes promote the regeneration of stem cells and haematopoiesis by secreting SCF. Nat Cell Biol. 2017 Aug 1 [cited 2020 Jul 13];19(8):891–903. Available from: https://pubmed.ncbi.nlm.nih.gov/28714970/. 10.1038/ncb3570 PMC5536858 28714970

[47] Burkhardt LM, Bucher CH, Löffler J, Rinne C, Duda GN, Geissler S, et al. The benefits of adipocyte metabolism in bone health and regeneration. Front Cell Dev Biol. 2023;11:1104709. 36895792 10.3389/fcell.2023.1104709 PMC9988968

[48] Thomas S, Fiebig JE, Kuhn EM, Mayer DS, Filbeck S, Schmitz W et al. Design of Glycoengineered IL-4 Antagonists Employing Chemical and Biosynthetic Glycosylation. ACS Omega. 2023 Jul 18 [cited 2026 Feb 6];8(28):24841. Available from: https://pmc.ncbi.nlm.nih.gov/articles/PMC10357448/. 10.1021/acsomega.3c00726 PMC10357448 37483220

[49] Josephson AM, Leclerc K, Remark LH, Lopeź EM, Leucht P. Systemic NF-κB-mediated inflammation promotes an aging phenotype in skeletal stem/progenitor cells. Aging (Albany NY). 2021 May 25 [cited 2024 Sep 25];13(10):13421–9. Available from: https://www.aging-us.com/article/203083. 10.18632/aging.203083 PMC8202837 34035186

[50] Bastidas-Coral AP, Bakker AD, Zandieh-Doulabi B, Kleverlaan CJ, Bravenboer N, Forouzanfar T et al. Cytokines TNF-α, IL-6, IL-17F, and IL-4 Differentially Affect Osteogenic Differentiation of Human Adipose Stem Cells. Stem Cells Int. 2016 Jan 1 [cited 2024 Sep 25];2016(1):1318256. Available from: https://onlinelibrary.wiley.com/doi/full/10.1155/2016/1318256. 10.1155/2016/1318256 PMC5030432 27667999

[51] Schlundt C, Fischer H, Bucher CH, Rendenbach C, Duda GN, Schmidt-Bleek K. The multifaceted roles of macrophages in bone regeneration: A story of polarization, activation and time. Acta Biomater. 2021;133:46–57. 33974949 10.1016/j.actbio.2021.04.052

[52] Kulakova K, Lawal TR, Mccarthy E, Floudas A. The Contribution of Macrophage Plasticity to Inflammatory Arthritis and Their Potential as Therapeutic Targets. Cells. 2024 Sep 1 [cited 2025 Nov 21];13(18). Available from: https://pubmed.ncbi.nlm.nih.gov/39329767/. 10.3390/cells13181586 PMC11430612 39329767

[53] Mantovani A, Biswas SK, Galdiero MR, Sica A, Locati M. Macrophage plasticity and polarization in tissue repair and remodelling. J Pathol. 2013 Jan 1 [cited 2024 Sep 25];229(2):176–85. Available from: https://onlinelibrary.wiley.com/doi/full/10.1002/path.4133. 10.1002/path.4133 23096265

[54] Németh K, Leelahavanichkul A, Yuen PST, Mayer B, Parmelee A, Doi K et al. Bone marrow stromal cells attenuate sepsis via prostaglandin E2–dependent reprogramming of host macrophages to increase their interleukin-10 production. Nat Med 2008 151. 2008 Nov 21 [cited 2024 Sep 25];15(1):42–9. Available from: https://www.nature.com/articles/nm.1905. 10.1038/nm.1905 PMC2706487 19098906

[55] Silfverswärd CJ, Penno H, Frost A, Nilsson O, Ljunggren Ö. Expression of markers of activity in cultured human osteoblasts: Effects of interleukin-4 and interleukin-13. Scand J Clin Lab Invest. 2010 Sep [cited 2024 Sep 25];70(5):338–42. Available from: https://www.tandfonline.com/doi/abs/10.3109/00365513.2010.488698. 10.3109/00365513.2010.488698 20509757

[56] Könnecke I, Serra A, El Khassawna T, Schlundt C, Schell H, Hauser A, et al. T and B cells participate in bone repair by infiltrating the fracture callus in a two-wave fashion. Bone. 2014;64:155–65. 24721700 10.1016/j.bone.2014.03.052

[57] Li Y, Toraldo G, Li A, Yang X, Zhang H, Qian WP, et al. B cells and T cells are critical for the preservation of bone homeostasis and attainment of peak bone mass in vivo. Blood. 2007;109(9):3839–48. 17202317 10.1182/blood-2006-07-037994 PMC1874582

[58] Manabe N, Kawaguchi H, Chikuda H, Miyaura C, Inada M, Nagai R et al. Connection Between B Lymphocyte and Osteoclast Differentiation Pathways. J Immunol. 2001 Sep 1 [cited 2024 Sep 25];167(5):2625–31. Available from: 10.4049/jimmunol.167.5.2625. 10.4049/jimmunol.167.5.2625 11509604

[59] Liu W, Zhang L, Xuan K, Hu C, Liu S, Liao L et al. Alpl prevents bone ageing sensitivity by specifically regulating senescence and differentiation in mesenchymal stem cells. Bone Res 2018 61. 2018 Sep 11 [cited 2024 Sep 26];6(1):1–15. Available from: https://www.nature.com/articles/s41413-018-0029-4. 10.1038/s41413-018-0029-4 PMC6131243 30210899

[60] Dong J, Zhao W, Zhao J, Chen J, Liu P, Zheng X et al. ALPL regulates pro-angiogenic capacity of mesenchymal stem cells through ATP-P2X7 axis controlled exosomes secretion. J Nanobiotechnology. 2024 Dec 1 [cited 2024 Sep 26];22(1):1–18. Available from: https://jnanobiotechnology.biomedcentral.com/articles/10.1186/s12951-024-02396-6. 10.1186/s12951-024-02396-6 PMC11015668 38609899

[61] Lin DPL, Dass CR. Transdifferentiation of adipocytes to osteoblasts: potential for orthopaedic treatment. J Pharm Pharmacol. 2018 Mar 1 [cited 2024 Sep 24];70(3):307–19. Available from: https://onlinelibrary.wiley.com/doi/full/10.1111/jphp.12862. 10.1111/jphp.12862 29365349

[62] Zhou BO, Yue R, Murphy MM, Peyer JG, Morrison SJ. Leptin-Receptor-Expressing Mesenchymal Stromal Cells Represent the Main Source of Bone Formed by Adult Bone Marrow. Cell Stem Cell. 2014;15(2):154–68. 24953181 10.1016/j.stem.2014.06.008 PMC4127103

[63] Philbrick KA, Wong CP, Branscum AJ, Turner RT, Iwaniec UT. Leptin stimulates bone formation in ob/ob mice at doses having minimal impact on energy metabolism. J Endocrinol. 2017 Mar 1 [cited 2024 Sep 26];232(3):461–74. Available from: https://joe.bioscientifica.com/view/journals/joe/232/3/461.xml. 10.1530/JOE-16-0484 PMC5288125 28057869

[64] Turner RT, Kalra SP, Wong CP, Philbrick KA, Lindenmaier LB, Boghossian S et al. Peripheral leptin regulates bone formation. J Bone Miner Res. 2013 Jan 1 [cited 2024 Sep 26];28(1):22–34. Available from: 10.1002/jbmr.1734. 10.1002/jbmr.1734 PMC3527690 22887758

[65] Chen Y, Wang H, Yang Q, Zhao W, Chen Y, Ni Q et al. Single-cell RNA landscape of the osteoimmunology microenvironment in periodontitis. Theranostics. 2022 [cited 2024 Sep 26];12(3):1074–96. Available from: https://www.thno.org//creativecommons.org/licenses/by/4.0/. 10.7150/thno.65694 PMC8771561 35154475

[66] Korbecki J, Gassowska-Dobrowolskaą M, Wójcik J, Szatkowska I, Barczak K, Chlubek M et al. The Importance of CXCL1 in Physiology and Noncancerous Diseases of Bone, Bone Marrow, Muscle and the Nervous System. Int J Mol Sci 2022, Vol 23, Page 4205. 2022 Apr 11 [cited 2024 Sep 26];23(8):4205. Available from: https://www.mdpi.com/1422-0067/23/8/4205/htm. 10.3390/ijms23084205 PMC9024980 35457023

[67] Bi J, Li Q, Yang Z, Cai L, Lv T, Yang X et al. CXCL2 Impairs Functions of Bone Marrow Mesenchymal Stem Cells and Can Serve as a Serum Marker in High-Fat Diet-Fed Rats. Front Cell Dev Biol. 2021 Jul 13 [cited 2024 Sep 26];9:687942. Available from: https://www.frontiersin.org. 10.3389/fcell.2021.687942 PMC8315099 34327200

[68] Jeong J, Kim YJ, Yoon SY, Kim YJ, Kim JH, Sohn KY et al. PLAG (1-Palmitoyl-2-Linoleoyl-3-Acetyl-rac-Glycerol) Modulates Eosinophil Chemotaxis by Regulating CCL26 Expression from Epithelial Cells. PLoS One. 2016 Mar 1 [cited 2024 Sep 26];11(3):e0151758. Available from: https://journals.plos.org/plosone/article?id=10.1371/journal.pone.0151758. 10.1371/journal.pone.0151758 PMC4807014 27010397

[69] Chu VT, Fröhlich A, Steinhauser G, Scheel T, Roch T, Fillatreau S et al. Eosinophils are required for the maintenance of plasma cells in the bone marrow. Nat Immunol 2011 122. 2011 Jan 9 [cited 2024 Sep 26];12(2):151–9. Available from: https://www.nature.com/articles/ni.1981. 10.1038/ni.1981 21217761

[70] Yang N, Liu Y. The Role of the Immune Microenvironment in Bone Regeneration. Int J Med Sci. 2021 [cited 2026 Feb 11];18(16):3697–707. Available from: http://www.medsci.org//creativecommons.org/licenses/by/4.0/. 10.7150/ijms.61080 PMC8579305 34790042

[71] Pajarinen J, Lin T, Gibon E, Kohno Y, Maruyama M, Nathan K et al. Mesenchymal stem cell-macrophage crosstalk and bone healing. Biomaterials. 2019 Mar 1 [cited 2026 Feb 11];196:80–9. Available from: 10.1097/00003086-198110000-00042. 10.1016/j.biomaterials.2017.12.025 PMC6028312 29329642

