# Supplementary material for: The importance of B cells for osteogenic homeostasis
Source: Cell Commun Signal. 2026 Aug 8;24:435. doi: 10.1186/s12964-026-03128-z (PMC13452110; doi:10.1186/s12964-026-03128-z)
Supplement: Supplementary file 1 [file 12964_2026_3128_MOESM1_ESM.docx]

**Supplementary Material:**

**Figure S 1: Flow cytomtric plots of the immune cell subsets analysed after isolation.** A) Analysis of the PBMC isolate. B) Analysis of the T cell isolate. C) Analaysis of the B cell isolate.

**Figure S 2 Flow cytomatic data of unactivated and activated PBMCs (A), T cells (B), B cells (C) and PBMCs of the Mixed lymphocyte reaction (D).**

**Figure S 3:** **Alizarin Red and Nile red staining during osteogenic or adipogenic differentiation of MSCs stimulated with different immune-conditioned media.** A) Quantification of alizarin red staining after stimulated osteogenic differentiation normalized to that of the control medium (dotted line). B) Quantification of nile red staining after stimulated adipogenic differentiation normalized to that of the control medium (dotted line). ICM MLR = mixed lymphocyte reaction immune-conditioned medium, ICM PBMC = activated peripheral blood mononuclear cell immune-conditioned medium, ICM T cell = activated T cell-immune-conditioned medium, ICM B cell = activated B cell immune-conditioned medium. The dots and error bars show the means of the technical replicates of each donor and the standard deviation. n=6.
